# Effect of Soy Isoflavones on Measures of Estrogenicity: A Systematic Review and Meta-Analysis of Randomized Controlled Trials

**DOI:** 10.1016/j.advnut.2024.100327

**Published:** 2024-10-20

**Authors:** Gabrielle Viscardi, Songhee Back, Amna Ahmed, Shuting Yang, Sonia Blanco Mejia, Andreea Zurbau, Tauseef A Khan, Amanda Selk, Mark Messina, Cyril WC Kendall, David JA Jenkins, John L Sievenpiper, Laura Chiavaroli

**Affiliations:** 1 Department of Nutritional Sciences, Temerty Faculty of Medicine, University of Toronto, Toronto, Ontario, Canada; 2 Toronto 3D Knowledge Synthesis and Clinical Trials Unit, Clinical Nutrition and Risk Factor Modification Centre, St. Michael’s Hospital, Toronto, Ontario, Canada; 3 Department of Medicine, Temerty Faculty of Medicine, University of Toronto, Toronto, Ontario, Canada; 4 Department of Gynecology, Women’s College Hospital, Toronto, Ontario, Canada; 5 Department of Obstetrics and Gynaecology, Mount Sinai Hospital (Toronto), Sinai Health, Toronto, Ontario, Canada; 6 Department of Obstetrics & Gynaecology, University of Toronto, Toronto, Ontario, Canada; 7 Soy Nutrition Institute Global, Washington, DC, United States; 8 College of Pharmacy and Nutrition, University of Saskatchewan, Saskatoon, Saskatchewan, Canada; 9 Division of Endocrinology and Metabolism, Department of Medicine, St. Michael’s Hospital, Toronto, Ontario, Canada; 10 Li Ka Shing Knowledge Institute, St. Michael’s Hospital, Toronto, Ontario, Canada

**Keywords:** soy isoflavones, soy foods, postmenopause, women, endometrial thickness, vaginal maturation index, follicle-stimulating hormone, estradiol, meta-analysis, systematic review

## Abstract

Despite recommendations to increase plant food consumption for public and planetary health and the role that soy foods can play in plant-predominant diets, controversies around the effects of soy foods, especially soy isoflavones, are a barrier to their intake. Given their cardioprotective effects and ability to alleviate menopausal symptoms, addressing these concerns is particularly relevant to women. This systematic review and meta-analysis of randomized controlled trials aimed to determine the effect of soy isoflavones on measures of estrogenicity in postmenopausal women. MEDLINE, Embase, and Cochrane Library were searched through August 2024 for randomized trials ≥3-mo investigating soy isoflavones compared with non-isoflavone controls in postmenopausal women. Outcomes included endometrial thickness (ET), vaginal maturation index (VMI), follicle-stimulating hormone (FSH), and estradiol. Independent authors extracted data and assessed risk of bias. Grading of Recommendations, Assessment, Development and Evaluation was used to assess certainty of evidence. We included 40 trials (52 trial comparisons, *n* = 3285) assessing the effect of a median reported dose of 75 mg/d of soy isoflavones in substitution for non-isoflavone controls over a median of 24 wk. Soy isoflavones had no statistically significant effect on any measure of estrogenicity; ET [mean difference, –0.22 mm (95% confidence interval, –0.45, 0.01 mm), *P*_MD_ = 0.059], VMI [2.31 (–2.14, 6.75), *P*_MD_ = 0.310], FSH [–0.02 IU/L (–2.39, 2.35 IU/L), *P*_MD_ = 0.987], and estradiol [1.61 pmol/L (–1.17, 4.38 pmol/L), *P*_MD_ = 0.256]. The certainty of evidence was high to moderate for all outcomes. Current evidence suggests that soy isoflavones do not exhibit estrogenic effects compared with non-isoflavone controls on 4 measures of estrogenicity in postmenopausal women. This synthesis supports that soy isoflavones likely act as selective estrogen receptor modulators, differing clinically from the hormone estrogen. Addressing public health concerns may promote soy foods as high-quality plant protein sources with low environmental impact and cost, particularly benefiting postmenopausal women and aligning with sustainable dietary patterns and guidelines.

This study was registered in PROSPERO as CRD42023439239.

**Statement of significance**

This systematic review and meta-analysis provides evidence that soy isoflavones results in no effects on 4 measures of estrogenicity in postmenopausal women related to the endothelium, supporting that soy isoflavones likely act as selective estrogen receptor modulators, differing clinically from the hormone estrogen. These findings help alleviate safety concerns and reinforce the potential of soy foods as high-quality plant proteins that can contribute to cardiovascular health, particularly in postmenopausal women.

## Introduction

Despite dietary recommendations to consume more plant foods for public and planetary health [[1], [2], [3], [4], [5]] and the role that soy foods can play in plant-predominant diets, controversies around the effects of soy foods and their components, especially soy isoflavones, are a barrier to their intake. The negative views of soy, including a worsening of the prognosis of women with estrogen-sensitive breast cancer [6], are predominately driven by the results of animal studies [[7], [8], [9], [10]]. These studies have limited implications for human health, in part because of the differences in metabolism of soy isoflavones between rodents and humans [[11], [12], [13]]. In contrast, human studies indicate soy has cardioprotective effects where soy has health claims for cholesterol and coronary artery disease risk reduction [[14], [15], [16]]. These potential benefits are particularly relevant to women in which cardiovascular disease (CVD), the leading cause of death in women [17], is underrecognized and undertreated [18,19]. Furthermore, there is also evidence that soy isoflavones reduce risk of breast [20], endometrial [21], and prostate [22] cancer, improve bone health [23] and memory [24], and alleviate menopausal symptoms [25]. Although menopause is sex-specific, we will retain use of the term women as it is used conventionally in studies and guidelines on menopause [26]. Because ≤80% of menopausal women experience moderate-to-severe vasomotor symptoms [27] and there are some cancer-related concerns with the use of hormone replacement therapy (HRT), soy isoflavones are an alternative of interest, as indicated by the 2023 position statement of The North American Menopause Society (NAMS) [26,28].

Even with the known benefits, there is still public concern about the estrogenicity of soy foods and isoflavones. Although isoflavones are commonly classified as phytoestrogens, they differ from the hormone estrogen at both the molecular and clinical levels. For example, isoflavones preferentially bind to estrogen receptor (ER)β in comparison with ERα, whereas estrogen binds with equal affinity to these receptors [29,30]. These receptors have different tissue distributions and when bound by ligands result in different and sometimes opposite physiological effects [31]. In general, activation of ERα and ERβ is seen as exerting proliferative and antiproliferative effects, respectively [31]. Their preferential binding may provide a conceptual basis for classifying isoflavones as selective estrogen receptor modulators (SERMs) [32].

Consumption of soy foods is extremely low in North Americans with only 3%–4% reporting consumption of a soy-containing product on any given day [33]. Because both national dietary guidance and cardiovascular clinical practice guidelines on nutrition therapy recommend to consume more plant foods [[1], [2], [3], [4], [5]], including soy foods, there is a public health need to better understand and characterize soy isoflavones to support intake. Addressing public concerns over the estrogenicity of soy foods will also support addressing the health equity gap in CVD as this concern is of particular relevance to menopausal women given the increase in CVD risk and vasomotor symptoms, which increased soy consumption may alleviate. We therefore undertook a systematic review and meta-analysis of randomized controlled trials in postmenopausal women to determine the effect of soy isoflavones on 4 measures of estrogenicity: endometrial thickness (ET), vaginal maturation index (VMI), follicle-stimulating hormone (FSH), and circulating estradiol.

## Methods

We followed the Cochrane Handbook for Systematic Reviews of Interventions (version 6.5) [34] for the conduct of our systematic review and meta-analysis and reported our results following the Preferred Reporting Items for Systematic Reviews and Meta-Analysis (PRISMA-Equity) guidelines [35]. The study protocol was registered on PROSPERO (CRD42023439239).

### Data sources and search strategy

We systematically searched MEDLINE, Embase, and the Cochrane Central Register of Controlled Studies from inception through August 1, 2024. Supplemental Tables 1 and 2 show the search strategy on the basis of the PICOTS framework without language restrictions. Validated filters from the Cochrane Handbook for Systematic Reviews of Interventions were applied to limit the database search to controlled studies [36]. Manual searches of the reference lists of included studies complemented the systematic search.

### Study selection

Three reviewers (AA, GV, and SB) examined studies retrieved from the search. We included randomized controlled feeding trials in postmenopausal women of all health backgrounds, with intervention periods ≥3 mo. We included studies that investigated the effect of isoflavones from soy (as either supplements or foods) compared with a suitable non-isoflavone-containing control (such as placebo capsules or soy protein nearly devoid of isoflavones) on measures of estrogenicity. As our objective was to target the results of our analyses to isoflavones from soy-based sources (soybeans primarily contain genistein and daidzein), we excluded non-soy sources of isoflavones as they may contain different forms of isoflavones (for example, red clover primarily contains biochanin A and formononetin). We also excluded studies of interventions containing no isoflavones. In reports containing >1 eligible trial comparison, we included all available trial comparisons.

### Data extraction and quality assessment

At least 2 independent reviewers (GV, SB, and AA) extracted relevant data from eligible studies. Relevant information included the number of participants, age, health status, years since last menses, study design, level of feeding control, intervention type, soy isoflavone reported dose, comparator, follow-up duration, energy balance, energy control, funding source, and outcome data. Authors were contacted for missing outcome data when it was indicated that relevant outcomes were measured but not reported. In the absence of numerical values for outcomes and inability to obtain the original data from authors, values were extracted from figures using Plot Digitizer where available [37].

Included studies were assessed for risk of bias (ROB) independently by ≥2 independent reviewers (GV, SB, and AA) with the Cochrane Risk of Bias V.2.0 tool [38]. Assessment was done across 6 domains of bias [randomization process, ROB arising from period or carryover effects (crossover studies only), deviations from intended interventions, missing outcome data, measurement of the outcome, and selection of the reported results]. The tool provides a judgment of “low risk of bias,” “some concerns,” or “high risk of bias” for each domain on the basis of the responses to signaling questions. An overall ROB was determined on the basis of the judgments from each domain. We resolved discrepancies in data extraction and ROB by consensus and review by the senior author (LC).

### Outcomes

The primary outcomes included 4 measures of estrogenicity: ET, VMI, FSH, and levels of estradiol. These measures were chosen because they are known to be affected by the hormone estrogen and were evaluated in many trials involving isoflavones. Mean differences (MDs) between the intervention and control arm and respective standard errors were extracted for each trial. If these were not provided, they were derived from available data using published formulas [34]. Mean pairwise difference in change-from-baseline values were preferred over end values. When median data was provided, they were converted to mean data with corresponding variances using methods developed by McGrath et al. [39]. When no variance data was available, the standard deviation of the MDs was borrowed from a trial similar in size, participants, and nature of intervention.

### Data synthesis and analysis

We used STATA software, version 18.0 (StataCorp) for all analyses. The principal effect measures were the mean pairwise differences in change from baseline (or alternatively, end differences) between the intervention arm providing the soy isoflavones and the comparator/control arm in each trial comparison (significance at *P*_MD_ < 0.05). Results are reported as MDs with 95% confidence intervals (95% CIs). Data were analyzed using the generic inverse variance method with DerSimonian and Laird random-effects model [40]. A fixed-effects model was used when the number of trial comparisons was <5 [41]. Paired analyses were applied to all crossover trials with the use of a within-individual correlation coefficient between treatment of 0.5 as described by Elbourne et al. [[42], [43], [44]]. To mitigate a unit-of-analysis error, when arms of trials with multiple intervention or control arms were used more than once, the corresponding sample size was divided by the number of times it was used for calculation of the standard error [45]. Each pairwise trial comparison was considered a separate trial for the purpose of this analysis.

Heterogeneity was assessed using the Cochran *Q* statistic and quantified using the *I*^2^ statistic [46]. We considered an *I*^2^ ≥ 50% and *P*_*Q*_ < 0.10 as evidence of substantial heterogeneity [47]. Sources of heterogeneity were explored by sensitivity and subgroup analyses. We conducted sensitivity analyses by influence analysis in which each trial was systematically removed from the meta-analysis with recalculation of the summary effect estimate. A trial whose removal explained the heterogeneity or changed the significance, direction, or magnitude [by more than the minimally important difference (MID) for harm set at 0.30 for ET, 3.8 for VMI, 7.4 for FSH and 5.2 for estradiol based on 10% of the baseline mean of 3.0 mm, 37.6, 7.4 IU/L, and 5.2 pmol/L, respectively] of the effect was considered an influential trial. To determine whether the overall results were robust to the use of different correlation coefficients in crossover trials, we also conducted sensitivity analyses using correlation coefficients of 0.25 and 0.75. We also performed sensitivity analyses using fixed-effects model. If ≥10 trials were available [48,49], we conduced subgroup analyses to explore sources of heterogeneity using meta-regression (*P*_*Q*_ < 0.05). A priori subgroup analyses were conducted by age, participant health status, baseline outcome level, years since last menses, soy isoflavone reported dose, intervention type, comparator, follow-up duration, study design, energy balance of the intervention relative to the basal diet (neutral, positive, and negative), level of energy control relative to the comparator (substitution, addition, subtraction, and ad libitum), feeding control (dietary advice, supplemented, and metabolic), funding, and ROB. Post hoc subgroup analyses were conducted by type of MD (change from baseline or end differences), baseline BMI, comparator by presence of soy protein, and by continent where the study was conducted. Meta-regression analyses were used to assess the significance of each subgroup categorically and when possible, continuously.

If ≥6 trial comparisons were available [50], dose–response analyses were performed using meta-regression to assess linear (by generalized least squares trend estimation models) and nonlinear spline curve modeling (by the MKSPLINE procedure with 3 knots [51]) dose–response gradients (*P* < 0.05).

If ≥10 trials were available [52], we assessed publication bias by inspection of contour-enhanced funnel plots and formal testing with Egger’s and Begg’s tests (*P* < 0.10) [[53], [54], [55]]. If there was evidence of publication bias, we adjusted for funnel plot asymmetry by imputing the missing trial data using the Duval and Tweedie trim-and-fill method and assessed for small study effects [56].

### Certainty of the evidence

The certainty of the evidence was assessed using the Grading of Recommendations, Assessment, Development and Evaluation (GRADE) approach [57] and software (GRADEpro V.3.2 [58]). Evidence was rated as high, moderate, low, or very low certainty. The included randomized trials were initially rated as high certainty by default and then downgraded or upgraded on the basis of prespecified criteria. Reasons for downgrading the evidence included ROB (Cochrane Risk of Bias V.2.0 tool [38]), inconsistency (substantial unexplained interstudy heterogeneity, *I*^2^ ≥ 50%, *P*_*Q*_ < 0.10), indirectness (absence or presence of factors limiting the generalizability of results), imprecision (95% CI for pooled effect estimates cross the MID for harm), and publication bias (significant evidence of small study effects). The reason for upgrading the evidence was the presence of a significant dose–response gradient [[59], [60], [61], [62], [63], [64], [65], [66]]. The importance of the magnitude of the pooled estimates was assessed using our prespecified MIDs and the effect size categories according GRADE guidance [[67], [68], [69], [70]] as follows: large effect (≥5× MID), moderate effect (≥2× MID), small important effect (≥1× MID), and trivial/unimportant effect (<1 MID).

## Results

### Search results

**FIGURE 1:**
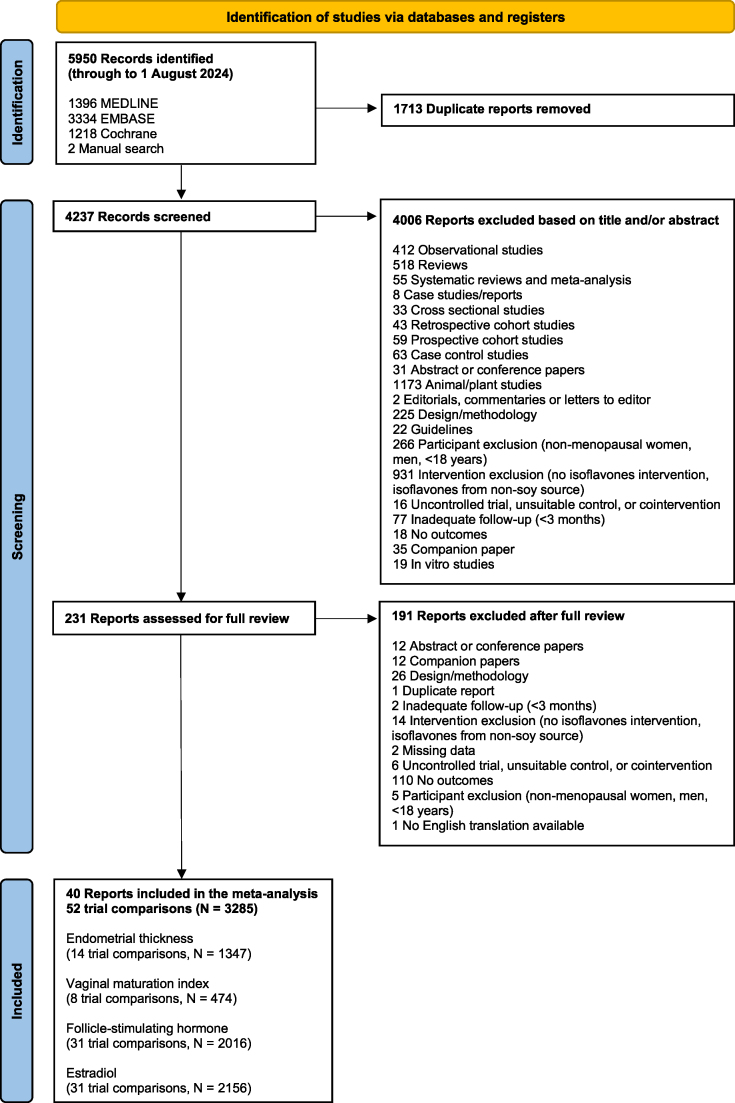
Flow of literature for the effect of soy isoflavones on measures of estrogenicity.

Figure 1 shows the flow of the literature review. We retrieved 5858 reports from databases and manual searches, 5636 of which were excluded based on the title or abstract. Of the 224 reports reviewed in full text, 40 reports of randomized trials (52 trial comparisons, *N* = 3285) met the eligibility criteria [[71], [72], [73], [74], [75], [76], [77], [78], [79], [80], [81], [82], [83], [84], [85], [86], [87], [88], [89], [90], [91], [92], [93], [94], [95], [96], [97], [98], [99], [100], [101], [102], [103], [104], [105], [106], [107], [108], [109], [110]].

### Trial characteristics

Table 1 and Supplemental Table 3 show the trial characteristics. Trial sizes ranged from a median of 50 participants (range: 18–245) for FSH to 65 (27–389) for ET. Participants were postmenopausal women with a median age of 54 (48–71) y for VMI, FSH, and estradiol and 55 (49–74) y for ET, without a diagnosis of a chronic disease, except for 1 trial involving participants with a history of breast cancer and another with insulin resistance. Years since last menses ranged from a median 5 (2–19) y for trials of FSH and estradiol to a median of 7 (2–24) y for trials of ET. Most trials were performed in North America (29%–38%) and Europe (10%–43%) and were parallel in design (ranging from 75 to 93% in trials of VMI and ET, respectively). Feeding control was mostly supplemented (88%–100%). The median dose of soy isoflavones reported ranged from 66 mg (36%–154) in trials of ET to 77 mg (40–600 mg) in trials of FSH. The intervention duration ranged from a median of 13 wk (12–104 wk) in trials of FSH to 24 wk (12–156 wk) for ET. Most trials were funded by agency sources (government, not-for-profit health agency, or university sources) (19%–43%), followed by both agency and industry sources (7%–39%). The comparators included placebo capsules of casein (2%), lactose (7%) and dextrin (6%), soy protein nearly devoid of isoflavones (4%), starch (5%), casein-based foods/beverages (10%), usual diet (5%), non-soy-based foods/beverages (2%), soy protein-based foods/beverages nearly devoid of isoflavones (8%), and milk protein-based foods/beverages (8%). Of comparators, most did not include soy protein (88%) whereas a few included soy proteins nearly devoid of isoflavones (12%).

**TABLE 1:** Summary of characteristics of included trial comparisons assessing the effect of soy isoflavones on outcome measures of estrogenicity^1^.

Trial characteristics	Endometrial thickness	Vaginal maturation index	Follicle-stimulating hormone	Estradiol
Trial comparisons (No)	14	8	31	31
Study size [median No (range) of participants]^2^	65 (27–389)	54 (18–142)	50 (18–245)	56 (18–245)
Age [years; median (range)]^3^	55 (49–74)	54 (49–57)	54 (48–71)	54 (48–71)
Health status (%; absence of disease: insulin resistance: history breast cancer)	93:0:7	100:0:0	97:0:3	94:3:3
Years since menopause [years; median (range)]^3^	7 (2–24)	7 (3–13)	5 (2–19)	5 (2–19)
Continent (No. of comparisons, and by country)	North America = 4 (USA = 4); South America = 3 (Brazil = 3); South Asia = 1 (India = 1); Europe = 6 (Finland = 1; Italy = 4; Sweden = 1)	North America = 3 (USA = 3); South America = 2 (Brazil = 2); South Asia = 1 (India = 1); Oceania = 1 (Australia = 1); Europe = 1 (Italy = 1)	North America = 12 (USA = 12); South America = 5 (Brazil = 4; Chile = 1); East Asia = 4 (China = 2; Japan = 1; South Korean = 1); South Asia = 4 (India = 4); Oceania = 3 (Australia = 3); Europe = 3 (Finland = 1; Italy = 1; Sweden = 1)	North America = 11 (USA = 11); South America = 5 (Brazil = 4; Chile = 1); East Asia = 9 (China = 4; Japan = 4; South Korean = 1); South Asia = 2 (India = 2); Europe = 4 (Finland = 1; Italy = 1; Spain = 1; Sweden = 1)
Study design (%; crossover: parallel)	7:93	25:75	13:87	13:87
Feeding control (%; supplemented: ad libitum)	93:7	88:12	100	100
Dose of isoflavones [mg; median (range)]	66 (36–154)	76 (47–200)	77 (40–600)	75 (40–200)
Follow-up duration [median No. (range) of weeks]	24 (12–156)	20 (12–104)	13 (12–104)	24 (12–104)
Funding sources (%; A: I: A, I: NR)^4^	43:36:7:14	38:25:37:0	19:23:32:26	29:16:39:16
Comparator (No. of comparisons)	Usual diet = 1; Placebo capsule = 4; Lactose capsule = 1; Soy protein capsule without isoflavone = 1; Calcium supplement = 2 Soy protein-based food/beverage without isoflavone = 1; Milk protein-based food/beverage = 3; Oatmeal beverage = 1	Usual diet = 1; Placebo capsule = 1; Lactose capsule = 1; Calcium supplement = 1; Wheat flour-based food/beverage = 1; Soy protein-based food/beverage without isoflavone = 2; Milk protein-based food/beverage = 1	Casein protein-based food/beverage = 6; Casein capsule = 1; Placebo capsule = 11 Lactose capsule = 2; Soy protein capsule without isoflavone = 1; Dextrin capsule = 1; Wheat flour-based food/beverage = 1; Starch capsule = 2; Non-soy-based food/beverage = 1; Soy protein-based food/beverage without isoflavone = 2; Milk protein-based food/beverage = 2; Oatmeal beverage = 1	Usual diet = 2; Casein protein-based food/beverage = 2; Casein capsule = 1; Placebo capsule = 11; Lactose capsule = 2; Soy protein capsule without isoflavones = 1; Dextrin capsule = 4; Starch capsule = 2; Non-soy-based food/beverage = 1; No capsule = 1; Soy protein-based food/beverage without isoflavone = 2; Milk protein-based food/beverage = 1; Oatmeal beverage = 1
Soy protein containing comparator (%; non-soy protein-containing: soy protein-containing	86:14	75:25	90:10	90:10
Intervention (No. of interventions)	Isoflavone capsule = 9; Soy protein-based beverage/food = 4; Isoflavone containing beverage = 1	Isoflavone capsule = 2; Soy protein powder = 1; Soy protein-based beverage/food = 5	Isoflavone capsule = 19; Soy protein powder = 1; Soy protein-based beverage/food = 9; Isoflavone containing beverage = 2	Isoflavone capsule = 24 Soy protein-based beverage/food = 6 Isoflavone containing beverage = 1
Setting (%; outpatient: inpatient)	100:0	100:0	100:0	100:0
Baseline BMI [kg/m^2^; median (range)]^3^	26.3 (24.9–29.1)	25.8 (25.2–29.0)	25.6 (22.6–29.1)	25.2 (21.1–29.1)
Baseline outcome [median (range)]^3^^,^^5^	3.1 (2.2–4.1)	41.8 (27.4–49.0)	76.0 (40.7–108.8)	44.2 (12.8–133.5)
Energy balance (%; neutral: positive: negative)^6^	64:36:0	25:75:0	65:35:0	77:23:0
Energy control (%; substitution: addition: subtraction)^7^	86:14:0	50:50:0	84:13:3	90:10:0
Type MD (%; CFB: ED)	93:7	100:0	94:6	90:10

Abbreviations: A, agency; CFB, change from baseline; ED, end difference; I, industry; MD, mean difference.

1 All numbers with the exception of baseline values were rounded to the nearest whole number to improve readability.

2 All sample sizes reflect participants included in the data analyzed.

3 Not all trials reported baseline values. Baseline values were not reported for: age (*n* = 6), years since menopause (*n* = 17), and baseline BMI (*n* = 8).

4 Agency funding is that from government, university, or not-for-profit sources. The majority of industry funding is that from trade organizations that obtain revenue from the sale of products.

5 Not all trials reported baseline outcome measures. Baseline outcome measures were not reported for: vaginal maturation index (*n* = 1).

6 Neutral energy balance refers to the maintenance of usual energy intake. Positive energy balance refers to a greater than normal energy intake. Negative energy balance refers to a deficit in normal energy intake.

7 Energy control refers to the energy intake of the intervention group compared with the control group where substitution refers to energy matched between intervention and comparator, addition refers to excess energy between intervention and comparator, and subtraction refers to deficit in energy between intervention and comparator.

### Risk of bias

Supplemental Figures 1 and 2 show a summary of the ROB assessments. Across outcomes, most trials were assessed as having low ROB in the randomization (86%–97%), missing (63%–87%), measurements (100%), and selection (71%–88%) domains, and some concerns in the deviations (35%–42%) domain. Fewer trials were assessed as having high ROB ranging from 3% in the randomization and measurements domains, to 16%–38% in the deviations domain, and 13%–38% in the missing domain. Most trials were judged overall as low (32%–39%) or some (25%–48%) concerns, with fewer as high ROB (19%–38%).

### Primary outcomes

**FIGURE 2:**
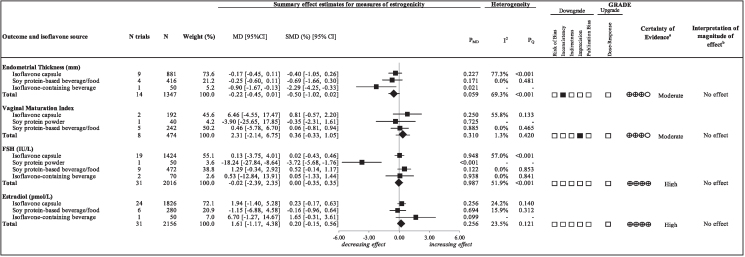
Summary plot of the effect of soy isoflavones on measures of estrogenicity in postmenopausal women. Data are weighted mean differences (95% CIs) using the generic inverse variance method modeled by random effects meta-analyses for summary effects of soy isoflavones on measures of estrogenicity. To allow the pooled effect estimates for each end point to be displayed on the same axis, mean differences were transformed to SMDs. Pseudo-95% CIs for each transformed SMD were derived directly from the original mean difference and 95% CIs. Between-study heterogeneity was assessed by the Cochran Q statistic, where *P*_Q_ < 0.100 is considered statistically significant, and quantified by the *I*^2^ statistic, where *I*^2^ ≥ 50% is considered evidence of substantial heterogeneity. The effects of total soy isoflavones are denoted by the effect estimates as diamonds. The effects of individual isoflavone sources are denoted by the effect estimates as squares. The Grading of Recommendations, Assessment, Development and Evaluation (GRADE) of randomized controlled trials are rated as “High” certainty of evidence and can be downgraded by 5 domains and upgraded by 1 domain. The white squares represent no downgrades, whereas filled black squares indicate a single downgrade or upgrades for each outcome. CI, confidence interval; ET, endometrial thickness; FSH, follicle-stimulating hormone; GRADE, Grading of Recommendations, Assessment, Development and Evaluation; MD, mean difference; MID, minimally important difference; N, number; ROB, risk of bias; SMD, standardized mean difference. ^a^ Because all included trials were randomized controlled trials, the certainty of the evidence was graded as high for all outcomes by default and then downgraded or upgraded on the basis of prespecified criteria. Criteria for downgrades included ROB (downgraded if the majority of trials were considered to be at high ROB); inconsistency (downgraded if there was substantial unexplained heterogeneity [*I*^2^ ≥ 50%, *P*_*Q*_ < 0.10]; indirectness (downgraded if there were factors absent or present relating to the participants, interventions, or outcomes that limited the generalizability of the results); imprecision (downgraded if the 95% CI crossed the MID for harm set at 0.30 for ET, 3.8 for VMI, 7.4 for FSH and 5.2 for estradiol based on 10% of the baseline mean of 3.0 mm, 37.6, 7.4 IU/L, and 5.2 pmol/L, respectively), and publication bias [downgraded if there is evidence of publication bias based on funnel plot asymmetry and/or significant Egger’s or Begg’s tests (*P* < 0.10) with confirmation by adjustment by Duval and Tweedie trim-and-fill analysis]. Criteria for upgrades included a significant dose–response gradient. ^b^For the interpretation of the magnitude, we used the MIDs (see a above) to assess the importance of magnitude of our point estimate using the effect size categories according to new GRADE guidance. We then used the MIDs to assess the importance of the magnitude of our point estimates using the effect size categories according GRADE guidance (54–57) as follows: large effect (≥5 MID); moderate effect (≥2 MID); small important effect (≥1 MID); and trivial/unimportant effect (<1 MID).

Figure 2 and Supplemental Figures 3–6 present the effect of soy isoflavones on ET, VMI, FSH, and estradiol. Soy isoflavones had no statistically significant effects on any of the measures of estrogenicity; ET (14 trials; MD: –0.22 mm; 95% CI: –0.45, 0.01 mm, *P*_MD_ = 0.059; substantial heterogeneity, *I*^2^ = 69.3%, *P*_Q_ < 0.001), VMI (8 trials; MD: 2.31; 95% CI: –2.14, 6.75, *P*_MD_ = 0.310; no substantial heterogeneity, *I*^2^ = 1.3%, *P*_Q_ = 0.420), FSH (31 trials; MD: –0.02 IU/L; 95% CI: –2.39, 2.35 IU/L, *P*_MD_ = 0.987; substantial heterogeneity, *I*^2^ = 51.9%, *P*_Q_ < 0.001), and estradiol (31 trials; MD: 1.61 pmol/L; 95% CI: –1.17, 4.38 pmol/L, *P*_MD_ = 0.256; no substantial heterogeneity, *I*^2^ = 23.5%, *P*_Q_ = 0.121).

### Adverse events and acceptability

Supplemental Table 4 presents the data reported in 5 trials on acceptability and 18 trials on adverse events. All trials reported data descriptively except for 1 trial. Of the 5 trials reporting acceptability, women in the intervention groups mainly reported a dislike for taste or volume of food, with Knight et al. [110] reporting a tendency to dislike the taste of the soy isoflavone beverage compared with control (*P* = 0.07). Among the 18 trials reporting adverse events, gastrointestinal upset was the most common reported symptom, where it was experienced to a similar extent in both those in the intervention and control groups.

### Sensitivity analyses

Supplemental Figures 7–10 present the individual trial influence analyses for each outcome. Removal of either Atteritano et al. [71], Kenny et al. (control + soy isoflavone) [77], Nahas et al. [84], or Upmalis et al. [93] resulted in a gain of significance for a decrease in ET. Removal of either Jassi et al. [76] or Kim et al. [79] provided a partial explanation of the evidence of substantial heterogeneity for FSH.

Supplemental Table 5 shows sensitivity analyses for the different correlation coefficients (0.25 and 0.75) used in paired analyses of crossover trials for each outcome. The use of these different correlation coefficients did not alter the direction, magnitude, or significance of the effect or evidence for heterogeneity.

Supplemental Figures 11–14 present the sensitivity analyses where fixed-effects models were used. The use of a fixed-effects model resulted in soy isoflavones showing a significant reduction on ET (14 trials; MD: –0.12 mm; 95% CI: –0.24, –0.01 mm, *P*_MD_ = 0.032; substantial heterogeneity, *I*^2^ = 69.34%, *P*_Q_ < 0.001).

### Subgroup analyses

Supplemental Figures 15–23 present the subgroup analyses and continuous meta-regression analyses for the effect of soy isoflavones on ET, FSH, and estradiol as there were ≥10 trial comparisons. There was significant effect modification by years since menopause (trials with median <5 y tending toward a reduction, whereas trials with a median ≥5 y tending toward an increase in FSH), continent (with neither continental subgroup showing statistical significance), and intervention type and energy control; however, the latter 2 were driven by 1 trial in which soy protein powder showed a greater reduction than other trials examining FSH. In continuous subgroup analyses, the follow-up duration was significant (β = 0.09 [0.00–0.18], *P* = 0.044). These subgroups partially explained evidence of substantial heterogeneity for FSH (residual *I*^2^ = 25%–48%). There was also significant effect modification by comparator for estradiol; however, this was driven by 1 trial where the comparator was soy protein capsules without isoflavones that showed an increase in estradiol.

### Dose–response analyses

Supplemental Figures 24–27 present linear and nonlinear dose–response analyses. There was no dose response for the effect of soy isoflavones on any measure of estrogenicity.

### Small-study effects

Supplemental Figures 28–30 present the contour-enhanced funnel plots and publication bias assessments for all outcomes with ≥10 trials available. There was no evidence of funnel plot asymmetry for any outcome. Note that publication bias was not assessed for VMI as <10 trial comparisons were available (*n* = 8).

### GRADE assessment

Figure 2 and Supplemental Table 6 present the GRADE assessments. The certainty of evidence for the effect of soy isoflavones was moderate for ET (no effect), owing to a downgrade for inconsistency because of unexplained heterogeneity, moderate for VMI (no effect), owing to a downgrade for imprecision, and high for FSH (no effect) and estradiol (no effect).

## Discussion

This systematic review and meta-analysis included 40 trials (52 trial comparisons) involving 3285 postmenopausal women, who were predominantly middle aged, without diagnosed chronic disease, and a median 5–6 y since last menses. We showed that the consumption of soy isoflavones does not affect 4 measures of estrogenicity. These measures were chosen because they are known to be affected by the hormone estrogen and were evaluated in many trials involving soy isoflavones. The median intervention duration ranged from 13 (FSH) to 24 wk (ET, estradiol). The lack of estrogenic effect was robust to sensitivity and subgroup analyses. The median dose of soy isoflavones reported ranged from 66 mg/d (range 36–154 mg) in trials of ET to 77 mg/d (40–600 mg) in trials of FSH.

### Findings in the context of literature

The lack of estrogenic effect on ET in the current analysis is consistent with a 2016 systematic review and meta-analysis of trials ≥3 mo [111], which examined the effects of isoflavones from red clover and soy, in peri- and postmenopausal women. They found there was no significant change in ET when all women were included in the analysis [23 trials, 2167 participants, standardized mean difference (SMD): –0.05, 95% CI: –0.23, 0.13, *P* = 0.60]. However, a daily dose of >54 mg isoflavones reported in trials decreased ET by 0.26 mm (10 trials, 984 participants, SMD: –0.26, 95% CI: –0.45, –0.07, *P* = 0.007). This finding suggests that higher isoflavone doses, by reducing ET, could potentially reduce the risk of developing endometrial cancer, a suggestion for which there is some epidemiological support [111]. Additionally, Li et al. [112] found that when trials were stratified according to study location, isoflavone supplementation significantly decreased ET by 0.23 mm in North American trials (*N* = 7726 participants, SMD: –0.23, 95% CI: –0.44, –0.01, *P* = 0.04), but tended to increase ET in Asian trials (*N* = 3, 128 participants, SMD: 0.23; 95% CI: –0.04, 0.50, *P* = 0.10). The nonsignificant increase in ET observed in Asian trials, albeit on the basis of only 3 trials, may result from ethnic differences in isoflavone metabolism wherein Asians are more likely to host intestinal bacteria that convert daidzein into equol [113], which has a much higher receptor binding affinity for both ERs than its parent isoflavone daidzein [114]. However, observational studies involving Asians show soy or isoflavone intake is associated with a decreased risk of endometrial cancer [21]. Our analyses did not demonstrate effect modification on ET by continent, where the lack of effect was consistent across trials from varying continents.

The lack of effect on circulating estradiol and FSH levels is consistent with the findings of a 2009 systematic review and meta-analysis of trials ≥4 wk [115]. In their meta-analysis, neither soy nor isoflavone consumption affected estradiol, estrone, FSH, or luteinizing hormone levels in pre- (6–11 trials per comparison) or postmenopausal (21 trials per comparison) women. No previously published meta-analysis has explored the effects of isoflavones on VMI. Although only 8 trials examined this endpoint, the lack of effect is consistent with the results of the other 3 measures of estrogenicity considered in the current analysis.

The lack of estrogenic effects of soy isoflavones on ET, VMI, FSH, and estradiol in the current analysis does not rule out these soybean constituents from exerting estrogen-like effects on other tissues and measures or endpoints. As noted previously, isoflavones are classified as SERMs. By definition, SERMs have tissue-specific effects. For example, tamoxifen exerts an antiestrogenic effect on breast tissue, but an estrogenic effect on endometrial tissue [32]. Therefore, the current analysis does not undermine findings that isoflavones alleviate menopausal symptoms [25], reduce bone loss [23], and improve memory [24] in postmenopausal women; effects thought to result from the interaction between isoflavones and ERs. Similarly, they do not rule out isoflavones from exerting estrogenic effects on breast tissue, although substantial clinical evidence indicates this is not the case [116,117]. Nor do they rule out isoflavones from having antiestrogenic effects. In premenopausal women, genistein (a soybean isoflavone accounting for ∼50% of total isoflavone content of the soybean [118]) has demonstrated inhibitory effects on endometrial hyperplasia, a precancerous condition indicated by an irregular thickening of the endometrial wall [119]. Furthermore, soy isoflavones have been shown to reduce the risk of breast cancer recurrence in postmenopausal women with estrogen-dependent cancer taking anastrozole, an estrogen-lowering therapy [120]. However, the findings of the current systematic review and meta-analysis serve to illustrate that isoflavones differ clinically from the hormone estrogen. This difference is evident when comparing the effect of soy isoflavones to that of HRTs (Supplemental Table 7). In systematic reviews and meta-analyses of the effect of HRTs on measures of estrogenicity, they demonstrate increases in ET, VMI, and estradiol and reductions in FSH, which contrast with the lack of effects of soy isoflavones observed in the present analysis. Furthermore, these differences were also observed in a head-to-head comparison between HRT with soy isoflavones [100]. This differentiation is important because safety concerns raised about isoflavones are based on their similarity to estrogen.

### Implications

Although most trials in the present analysis provided soy isoflavones as capsules, the results of these trials did not differ from those trials in which isoflavones were provided as soy protein-based beverages or foods (∼30%). This lack of difference by soy isoflavone source is not surprising because the absorption and metabolism of isoflavones from supplements is similar to that of isoflavones from foods [121]. However, because soy foods contain numerous biologically active components, whole soy foods may exert benefits beyond isoflavones alone on health outcomes, such as cholesterol for which soy has a health claim [[14], [15], [16]]. Furthermore, mean soy isoflavone intake in the included trials (median 66–77 mg/d reported intake) was higher than the typical isoflavone intake among older Japanese women (30–50 mg/d) [122,123] and the typical amount associated with a range of beneficial effects in observational studies where a systematic review and meta-analysis demonstrated that each 10 mg/d increment of soy isoflavone intake was significantly associated with a 4% lower risk of overall cancer incidence [22]. The intake of soy isoflavones per capita in the United States [[124], [125], [126]] and Europe [127,128] is no >6 mg/d and most likely fewer than 3 mg. Traditional Asian soy foods (for example, tofu, miso, and soymilk) contain ∼3.5 mg soy isoflavones/g protein; thus, a typical serving of tofu or soymilk that provides ∼8 g of protein may contain ∼28 mg soy isoflavones, whereas foods made from soybeans using concentrated sources of soy protein such as soy protein isolates or soy protein concentrates, where as much as 90% of isoflavone content can be lost during the processing of soybeans, may contain lower concentrations of isoflavones. Therefore, soy foods containing soy protein isolates or concentrates may not necessarily be rich sources of isoflavones. However, in the case where soy isoflavones may be present, the current study results allay negative estrogenic concerns. For those seeking to observe the benefit of soy isoflavones, such as reduced menopausal symptoms [25], purified soy isoflavone supplements, the predominant form assessed in the included trials, may be an ideal source. Overall, given the general low intake of soy foods, our results also support the consumption of soy foods as sources of high-quality plant proteins with low environmental impact and cost, in alignment with dietary guidelines [[1], [2], [3], [4], [5]].

The results of these data also support addressing the health equity gap in CVD as this concern is of particular relevance to menopausal women given the fact that as the leading cause of death in women [17] which is underrecognized and undertreated [18,19], encouraging an increased intake of soy foods can reduce cardiovascular disease risk, as evident in health claims [[14], [15], [16]].

With the vast majority of peri- and postmenopausal women experiencing vasomotor symptoms that significantly impact their quality of life [27], there is a great demand for effective treatment options. Both HRTs and soy isoflavones have shown evidence in ameliorating these symptoms [25,26]. The NAMS 2023 position statement recognizes soy isoflavones as an alternative therapy [26]. Regarding HRTs, the position statement highlights that there has been a significant decline in the use of HRTs following the publication of the Women’s Health Initiative [26], which showed an association between HRT use and increased risk of estrogen-related cancers [28]. Our current findings support soy isoflavones as an alternative therapy, as we did not show estrogenic effects on ET, VMI, FSH, and estradiol, reinforcing previous research demonstrating that soy isoflavone intake is associated with a reduction in the risk of estrogenic-related cancers [20,21].

### Strengths and limitations

The strengths of the analyses include a comprehensive identification of all eligible studies resulting from a rigorous search and selection strategy; the inclusion of primarily high-quality trials providing the highest protection against bias; the use of intention-to-treat data, when available, providing more conservative pooled estimates [129], and using the GRADE approach to assess the overall certainty of evidence.

Limitations of the analysis include the evidence indicating serious inconsistency for the effect of soy isoflavones on ET and serious imprecision in the pooled estimate for VMI where the 95% CIs were wide and could not rule out evidence of the effect. Although the present analyses cannot necessarily be extrapolated to tissues and endpoints not examined in the current analysis, they do address the 4 measures of estrogenicity related to endometrial tissues.

Weighing these strengths and limitations, we graded the certainty in the evidence as high for FSH and estradiol and moderate for ET and VMI.

In conclusion, our synthesis demonstrates that in postmenopausal women, consumption of soy isoflavones results in no effects on 4 measures of estrogenicity, ET, VMI, FSH, and estradiol. Certainty in the evidence was high for FSH and estradiol and moderate for ET and VMI. The main sources of uncertainty, inconsistency for ET, and imprecision for VMI should be considered by future large high-quality trials. Despite their common classification as phytoestrogens, the results of this analysis provide a strong rationale for not assuming that soy isoflavones will exert health effects similar to the hormone estrogen. Addressing public health concerns around soy foods may support their intake as high-quality plant protein foods with low environmental impact and cost, aligning with dietary guidelines.

## Author contributions

The authors’ contributions were as follows – LC, AS, MM, CWCK, DJAJ, JLS: designed research (project conception, development of overall research plan, and study oversight); GV, SB, AA, SY: conducted the research (hands-on conduct of the experiments and data collection); GV, SB, TAK, AZ: analyzed data or performed statistical analysis; LC: wrote the paper, had the primary responsibility for the final content, took the responsibility for the integrity of the data and the accuracy of the data analysis, and supervised the study; and all the authors read and approved the final manuscript. The corresponding author attests that all listed authors meet authorship criteria and that no others meeting the criteria have been omitted.

## Funding

This work was supported by the United Soybean Board (the United States Department of Agriculture soy check-off program) and the Canadian Institutes of Health Research (funding reference number, 129920) through the Canada-wide Human Nutrition Trialists’ Network (NTN). The Diet, Digestive tract, and Disease (3D) Centre, funded through the Canada Foundation for Innovation and the Ministry of Research and Innovation’s Ontario Research Fund, provided the infrastructure for the conduct of this work. GV was funded by a CIHR Canada Graduate Scholarship and Toronto 3D Summer Scholarship award. SB was funded by an Undergraduate Student Research Program scholarship. AA was funded by a Charles Hollenburg Summer Scholarship. AZ was funded by a Toronto 3D Postdoctoral Fellowship Award. LC was funded by a Toronto 3D New Investigator Award. None of the sponsors had any role in study design; in the collection, analysis, and interpretation of data; in the writing of the report; and in the decision to submit the article for publication. But 1 of the co-authors, Mark Messina, who was not involved in data collection or analysis, is the Director of Nutrition Science and Research at the Soy Nutrition Institute Global, an organization that receives partial funding from the principal funder, the United Soybean Board (USB).

## Data availability

Data described in the manuscript, code book, and analytic code will be made available upon request pending.

## Conflict of interest

AZ is a part-time research associate at INQUIS Clinical Research Ltd, a contract research organization, and has received consulting fees from Glycemic Index Foundation. TAK has received research support from the Canadian Institutes of Health Research (CIHR), the International Life Science Institute (ILSI), and National Honey Board. He has been an invited speaker at the Calorie Control Council Annual meeting for which he has received an honorarium. He has received funding from the Toronto 3D Knowledge Synthesis and Clinical Trials foundation. MM was employed by the Soy Nutrition Institute Global, an organization that receives funding from the United Soybean Board (USB) and from members involved in the soy industry. CWCK has received grants or research support from the Advanced Food Materials Network, Agriculture and Agri-Foods Canada (AAFC), Almond Board of California, Barilla, Canadian Institutes of Health Research (CIHR), Canola Council of Canada, International Nut and Dried Fruit Council, International Tree Nut Council Research and Education Foundation, Loblaw Brands Ltd, the Peanut Institute, Pulse Canada, and Unilever. He has received in-kind research support from the Almond Board of California, Barilla, California Walnut Commission, Kellogg Canada, Loblaw Companies, Nutrartis, Quaker (PepsiCo), the Peanut Institute, Primo, Unico, Unilever, and WhiteWave Foods/Danone. He has received travel support and/or honoraria from the Barilla, California Walnut Commission, Canola Council of Canada, General Mills, International Nut and Dried Fruit Council, International Pasta Organization, Lantmannen, Loblaw Brands Ltd., Nutrition Foundation of Italy, Oldways Preservation Trust, Paramount Farms, the Peanut Institute, Pulse Canada, Sun-Maid, Tate & Lyle, Unilever, and White Wave Foods/Danone. He has served on the scientific advisory board for the International Tree Nut Council, International Pasta Organization, McCormick Science Institute, and Oldways Preservation Trust. He is a founding member of the International Carbohydrate Quality Consortium (ICQC), Executive Board Member of the Diabetes and Nutrition Study Group (DNSG) of the European Association for the Study of Diabetes (EASD), is on the Clinical Practice Guidelines Expert Committee for Nutrition Therapy of the EASD, and is a Director of the Toronto 3D Knowledge Synthesis and Clinical Trials foundation. DJAJ has received research grants from Saskatchewan & Alberta Pulse Growers Associations, the Agricultural Bioproducts Innovation Program through the Pulse Research Network, the Advanced Foods and Material Network, Loblaw Companies Ltd., Unilever Canada and Netherlands, Barilla, the Almond Board of California, Agriculture and Agri-food Canada, Pulse Canada, Kellogg’s Company, Canada, Quaker Oats, Canada, Procter & Gamble Technical Centre Ltd., Bayer Consumer Care, Springfield, NJ, Pepsi/Quaker, International Nut & Dried Fruit Council (INC), Soy Foods Association of North America, the Coca-Cola Company (investigator initiated, unrestricted grant), Solae, Haine Celestial, the Sanitarium Company, Orafti, the International Tree Nut Council Nutrition Research and Education Foundation, the Peanut Institute, Soy Nutrition Institute (SNI), the Canola and Flax Councils of Canada, the Calorie Control Council, the Canadian Institutes of Health Research (CIHR), the Canada Foundation for Innovation (CFI), and the Ontario Research Fund (ORF). He has received in-kind supplies for trials as a research support from the Almond Board of California, Walnut Council of California, the Peanut Institute, Barilla, Unilever, Unico, Primo, Loblaw Companies, Quaker (Pepsico), Pristine Gourmet, Bunge Limited, Kellogg Canada, and WhiteWave Foods. He has been on the speaker’s panel, served on the scientific advisory board and/or received travel support and/or honoraria from Nutritional Fundamentals for Health (NFH)-Nutramedica, Saint Barnabas Medical Center, The University of Chicago, 2020 China Glycemic Index (GI) International Conference, Atlantic Pain Conference, Academy of Life Long Learning, the Almond Board of California, Canadian Agriculture Policy Institute, Loblaw Companies Ltd, the Griffin Hospital (for the development of the NuVal scoring system), the Coca-Cola Company, Epicure, Danone, Diet Quality Photo Navigation (DQPN), Better Therapeutics (FareWell), Verywell, True Health Initiative (THI), Heali AI Corp, Institute of Food Technologists (IFT), Soy Nutrition Institute (SNI), Herbalife Nutrition Institute (HNI), Saskatchewan & Alberta Pulse Growers Associations, Sanitarium Company, Orafti, the International Tree Nut Council Nutrition Research and Education Foundation, the Peanut Institute, Herbalife International, Pacific Health Laboratories, Barilla, Metagenics, Bayer Consumer Care, Unilever Canada and Netherlands, Solae, Kellogg, Quaker Oats, Procter & Gamble, Abbott Laboratories, Dean Foods, the California Strawberry Commission, Haine Celestial, PepsiCo, the Alpro Foundation, Pioneer Hi-Bred International, DuPont Nutrition and Health, Spherix Consulting and WhiteWave Foods, the Advanced Foods and Material Network, the Canola and Flax Councils of Canada, Agri-Culture and Agri-Food Canada, the Canadian Agri-Food Policy Institute, Pulse Canada, the Soy Foods Association of North America, the Nutrition Foundation of Italy (NFI), Nutra-Source Diagnostics, the McDougall Program, the Toronto Knowledge Translation Group (St. Michael's Hospital), the Canadian College of Naturopathic Medicine, The Hospital for Sick Children, the Canadian Nutrition Society (CNS), the American Society of Nutrition (ASN), Arizona State University, Paolo Sorbini Foundation, the Institute of Nutrition, Metabolism and Diabetes, the Lawson Centre Nutrition Digital Series, and the 19^th^ Annual Stare-Hegsted Lecture. He received an honorarium from the United States Department of Agriculture to present the 2013 W.O. Atwater Memorial Lecture. He received the 2013 Award for Excellence in Research from the International Nut and Dried Fruit Council. He received funding and travel support from the Canadian Society of Endocrinology and Metabolism to produce mini cases for the Canadian Diabetes Association (CDA). He is a member of the International Carbohydrate Quality Consortium (ICQC). His wife, Alexandra L Jenkins, is a director and partner of INQUIS Clinical Research for the Food Industry, his 2 daughters, Wendy Jenkins and Amy Jenkins, have published a vegetarian book that promotes the use of the foods described here, The Portfolio Diet for Cardiovascular Risk Reduction (Academic Press/Elsevier 2020 ISBN:978-0-12-810510-8), and his sister, Caroline Brydson, received funding through a grant from the St. Michael’s Hospital Foundation to develop a cookbook for one of his studies. He is also a vegan. JLS has received research support from the Canadian Foundation for Innovation, Ontario Research Fund, Province of Ontario Ministry of Research and Innovation and Science, Canadian Institutes of health Research (CIHR), Diabetes Canada, American Society for Nutrition (ASN), International Nut and Dried Fruit Council (INC) Foundation, National Honey Board (U.S. Department of Agriculture [USDA] honey “Checkoff” program), Institute for the Advancement of Food and Nutrition Sciences (IAFNS; formerly ILSI North America), Pulse Canada, Quaker Oats Center of Excellence, The United Soybean Board (USDA soy “Checkoff” program), Protein Industries Canada (a Government of Canada Global Innovation Clusters), The Tate and Lyle Nutritional Research Fund at the University of Toronto, The Glycemic Control and Cardiovascular Disease in Type 2 Diabetes Fund at the University of Toronto (a fund established by the Alberta Pulse Growers), The Plant Protein Fund at the University of Toronto (a fund which has received contributions from IFF), and The Nutrition Trialists Network Research Fund at the University of Toronto (a fund which has received donations from the Calorie Control Council, Physicians Committee for Responsible Medicine, and vegan grants through the Karuna Foundation). He has received food donations to support randomized controlled trials from the Almond Board of California, California Walnut Commission, Peanut Institute, Barilla, Unilever/Upfield, Unico/Primo, Loblaw Companies, Quaker, Kellogg Canada, Danone, Nutrartis, Soylent, and Dairy Farmers of Canada. He has received travel support, speaker fees and/or honoraria from ASN, Danone, Dairy Farmers of Canada, FoodMinds LLC, Nestlé, Abbott, General Mills, Nutrition Communications, International Food Information Council (IFIC), Calorie Control Council, International Sweeteners Association, International Glutamate Technical Committee, Arab Beverages Association, and Phynova. He has or has had ad hoc consulting arrangements with Perkins Coie LLP, Tate & Lyle, Inquis Clinical Research, Ingredion, and Brightseed. He is a former member of the European Fruit Juice Association Scientific Expert Panel and a former member of the Soy Nutrition Institute (SNI) Scientific Advisory Committee. He is on the Clinical Practice Guidelines Expert Committees of Diabetes Canada, European Association for the study of Diabetes (EASD), Canadian Cardiovascular Society (CCS), and Obesity Canada/Canadian Association of Bariatric Physicians and Surgeons. He serves as an unpaid member of the Board of Trustees of IAFNS and formerly served as an unpaid scientific advisor for the Carbohydrates Committee of IAFNS. He is a Director at Large of the Canadian Nutrition Society (CNS), a founding member of the International Carbohydrate Quality Consortium (ICQC), an Executive Board Member of the Diabetes and Nutrition Study Group (DNSG) of the EASD, and a director of the Toronto 3D Knowledge Synthesis and Clinical Trials foundation. His spouse is an employee of AB InBev. LC has received research support from Protein Industries Canada (a Government of Canada Global Innovation Clusters), Alberta Pulse Growers, The United Soybean Board (USDA soy “Checkoff” program), and the Canadian Institutes of Health Research (CIHR). All other authors report no conflicts of interest to disclose.
