# Supplementary material for: Effect of Soy Isoflavones on Measures of Estrogenicity: A Systematic Review and Meta-Analysis of Randomized Controlled Trials
Source: Adv Nutr. 2024 Oct 20;16(1):100327. doi: 10.1016/j.advnut.2024.100327 (PMC11784794; doi:10.1016/j.advnut.2024.100327)
Supplement: multimedia component 1 [file mmc1.docx]

**Supplementary Materials**

Re: Viscardi et al. Effect of soy isoflavones on measures of estrogenicity: A systematic review and meta-analysis of randomized controlled trials.

Table of Contents

[**Supplementary Table 1.** Search strategy for randomized controlled trials assessing the effect of soy isoflavones on measures of estrogenicity 4](#_Toc153356541)

[**Supplementary Table 2.** PICOTS framework of the search strategy 7](#_Toc153356542)

[**Supplementary Table 3.** Table of characteristics of the randomized controlled trials assessing the effect of soy isoflavones on measures of estrogenicity 8](#_Toc153356543)

[**Supplementary Table 4.** Assessment of acceptability and adverse events* 14](#_Toc153356544)

[**Supplementary Table 5.** Sensitivity analyses of the use of correlation coefficients of 0.25 and 0.75 for crossover trials in the primary analysis of the effect of soy isoflavones on measures of estrogenicity 16](#_Toc153356545)

[**Supplementary Table 6.** GRADE assessment of study quality 17](#_Toc153356546)

[**Supplementary Table 7.** Effects of hormone replacement therapy on estrogen-related intermediate outcomes compared to soy isoflavones 18](#_Toc153356547)

[**Supplementary Figure 1.** Risk of bias proportion graph for the effect of soy isoflavones on measures of estrogenicity in parallel trials 19](#_Toc153356548)

[**Supplementary Figure 2.** Risk of bias proportion graph for the effect of soy isoflavones on measures of estrogenicity in crossover trials 21](#_Toc153356549)

[**Supplementary Figure 3.** Forest plot of randomized controlled trials of the effect of soy isoflavones on endometrial thickness 23](#_Toc153356550)

[**Supplementary Figure 4.** Forest plot of randomized controlled trials of the effect of soy isoflavones on vaginal maturation index 24](#_Toc153356551)

[**Supplementary Figure 5.** Forest plot of randomized controlled trials of the effect of soy isoflavones on follicle-stimulating hormone 25](#_Toc153356552)

[**Supplementary Figure 6.** Forest plot of randomized controlled trials of the effect of soy isoflavones on estradiol 27](#_Toc153356553)

[**Supplementary Figure 7.** Sensitivity analysis of the systematic removal of each trial for the effect of soy isoflavones on endometrial thickness 29](#_Toc153356554)

[**Supplementary Figure 8.** Sensitivity analysis of the systematic removal of each trial for the effect of soy isoflavones on vaginal maturation index 30](#_Toc153356555)

[**Supplementary Figure 9.** Sensitivity analysis of the systematic removal of each trial for the effect of soy isoflavones on follicle-stimulating hormone 31](#_Toc153356556)

[**Supplementary Figure 10.** Sensitivity analysis of the systematic removal of each trial for the effect of soy isoflavones on estradiol 32](#_Toc153356557)

[**Supplementary Figure 11.** Sensitivity analyses with the use of fixed effects models for the effect of soy isoflavones on endometrial thickness 33](#_Toc153356558)

[**Supplementary Figure 12.** Sensitivity analyses with the use of fixed effects models for the effect of soy isoflavones on vaginal maturation index 34](#_Toc153356559)

[**Supplementary Figure 13.** Sensitivity analyses with the use of fixed effects models for the effect of soy isoflavones on follicle-stimulating hormone 35](#_Toc153356560)

[**Supplementary Figure 14.** Sensitivity analyses with the use of fixed effects models for the effect of soy isoflavones on estradiol 37](#_Toc153356561)

[**Supplementary Figure 15 (1 of 3).** Subgroup analyses for the effect of soy isoflavones on endometrial thickness* 39](#_Toc153356562)

[**Supplementary Figure 15 (2 of 3).** Subgroup analyses for the effect of soy isoflavones on endometrial thickness* 41](#_Toc153356563)

[**Supplementary Figure 15 (3 of 3).** Subgroup analyses for the effect of soy isoflavones on endometrial thickness 42](#_Toc153356564)

[**Supplementary Figure 16 (1 of 3).** Subgroup analyses for the effect of soy isoflavones on follicle-stimulating hormone* 43](#_Toc153356565)

[**Supplementary Figure 16 (2 of 3).** Subgroup analyses for the effect of soy isoflavones on follicle-stimulating hormone* 45](#_Toc153356566)

[**Supplementary Figure 16 (3 of 3).** Subgroup analyses for the effect of soy isoflavones on follicle-stimulating hormone 46](#_Toc153356567)

[**Supplementary Figure 17 (1 of 3).** Subgroup analyses for the effect of soy isoflavones on estradiol* 47](#_Toc153356568)

[**Supplementary Figure 17 (2 of 3).** Subgroup analyses for the effect of soy isoflavones on estradiol* 49](#_Toc153356569)

[**Supplementary Figure 17 (3 of 3).** Subgroup analyses for the effect of soy isoflavones on estradiol 50](#_Toc153356570)

[**Supplementary Figure 18.** Risk of bias subgroup analyses for the effect of soy isoflavones on endometrial thickness 51](#_Toc153356571)

[**Supplementary Figure 19.** Risk of bias subgroup analyses for the effect of soy isoflavones on follicle-stimulating hormone 52](#_Toc153356572)

[**Supplementary Figure 20.** Risk of bias subgroup analyses for the effect of soy isoflavones on estradiol 53](#_Toc153356573)

[**Supplementary Figure 21.** Continuous meta-regression analysis for the effect of soy isoflavones on endometrial thickness* 54](#_Toc153356574)

[**Supplementary Figure 22.** Continuous meta-regression analysis for the effect of soy isoflavones on follicle-stimulating hormone* 55](#_Toc153356575)

[**Supplementary Figure 23.** Continuous meta-regression analysis for the effect of soy isoflavones on estradiol* 56](#_Toc153356576)

[**Supplementary Figure 24.** Linear and non-linear meta-regression analysis for the effect of soy isoflavones on endometrial thickness 57](#_Toc153356577)

[**Supplementary Figure 25.** Linear and non-linear meta-regression analysis for the effect of soy isoflavones on vaginal maturation index 58](#_Toc153356578)

[**Supplementary Figure 26.** Linear and non-linear meta-regression analysis for the effect of soy isoflavones on follicle-stimulating hormone 59](#_Toc153356579)

[**Supplementary Figure 27.** Linear and non-linear meta-regression analysis for the effect of soy isoflavones on estradiol 60](#_Toc153356580)

[**Supplementary Figure 28.** Publication bias funnel plots for the effect of soy isoflavones on endometrial thickness 61](#_Toc153356581)

[**Supplementary Figure 29.** Publication bias funnel plots for the effect of soy isoflavones on follicle-stimulating hormone 62](#_Toc153356582)

[**Supplementary Figure 30.** Publication bias funnel plots for the effect of soy isoflavones on estradiol 63](#_Toc153356583)

# **Supplementary Table 1.** Search strategy for randomized controlled trials assessing the effect of soy isoflavones on measures of estrogenicity

| **MEDLINE** | **EMBASE** | **The Cochrane Library of Controlled Trials** |
| --- | --- | --- |
|  |  |  |
| 1. soy.mp. 2. Isoflavon*.mp. 3. exp Isoflavones/ 4. exp Soybeans/ 5. soy protein.mp. 6. exp Soybean Proteins/ 7. exp Soy Foods/ 8. exp Soy Milk/ 9. (soy adj3 milk).mp. 10. soy food$.mp. 11. (soy adj3 beverage$).mp. 12. isolated soy protein.mp. 13. soy protein isolate*.mp. 14. soya.mp. 15. Phytoestrogens.mp. 16. Tofu*.mp. 17. Natto*.mp. 18. Tempeh*.mp. 19. Miso*.mp. 20. exp tofu/ 21. soybeans.mp. 22. (soy adj3 bean$).mp. 23. exp Genistein/ 24. genistein.mp. 25. genistin.mp. 26. daidzein.mp. 27. daidzin.mp. 28. exp Equol/ 29. equol.mp. 30. glycitein.mp. 31. glycitin.mp. 32. 1 or 2 or 3 or 4 or 5 or 6 or 7 or 8 or 9 or 10 or 11 or 12 or 13 or 14 or 15 or 16 or 17 or 18 or 19 or 20 or 21 or 22 or 23 or 24 or 25 or 26 or 27 or 28 or 29 or 30 or 31 33. FSH.mp. 34. exp Follicle Stimulating Hormone/ 35. follicle stimulating hormone.mp. 36. endometrial.mp. 37. exp Endometrium/ 38. endometrium.mp. 39. vaginal maturation.mp. 40. vaginal maturation index.mp. 41. E2.mp. 42. exp Estradiol/ 43. estradiol.mp. 44. oestradiol.mp. 45. exp Estrogens/ 46. estrogen.mp. 47. oestrogen.mp. 48. 33 or 34 or 35 or 36 or 37 or 38 or 39 or 40 or 41 or 42 or 43 or 44 or 45 or 46 or 47 49. 32 and 48 50. randomized controlled trial.pt. 51. controlled clinical trial.pt. 52. randomized.ab. 53. placebo.ab. 54. clinical trials as topic.sh. 55. randomly.ab. 56. trial.ti. 57. 50 or 51 or 52 or 53 or 54 or 55 or 56 58. 49 and 57 | 1. soy.mp. 2. Isoflavon*.mp. 3. exp Isoflavones/ 4. exp Soybeans/ 5. soy protein.mp. 6. exp Soybean Proteins/ 7. exp Soy Foods/ 8. exp Soy Milk/ 9. (soy adj3 milk).mp. 10. soybeans.mp. 11. (soy adj3 bean$).mp. 12. soy food$.mp. 13. (soy adj3 beverage$).mp. 14. isolated soy protein.mp. 15. soy protein isolate*.mp. 16. soya.mp. 17. Phytoestrogens.mp. 18. Tofu*.mp. 19. Natto*.mp. 20. Tempeh*.mp. 21. Miso*.mp. 22. exp tofu/ 23. exp fermented soybean/ 24. exp genistein/ 25. genistein.mp. 26. exp genistin/ 27. genistin.mp. 28. exp daidzein/ 29. daidzein.mp. 30. exp daidzin/ 31. daidzin.mp. 32. exp equol/ 33. equol.mp. 34. exp glycitein/ 35. glycitein.mp. 36. glycitin.mp. 37. 1 or 2 or 3 or 4 or 5 or 6 or 7 or 8 or 9 or 10 or 11 or 12 or 13 or 14 or 15 or 16 or 17 or 18 or 19 or 20 or 21 or 22 or 23 or 24 or 25 or 26 or 27 or 28 or 29 or 30 or 31 or 32 or 33 or 34 or 35 or 36 38. FSH.mp. 39. follicle stimulating hormone.mp. 40. exp endometrial thickness/ 41. endometrial.mp. 42. exp endometrium/ 43. endometrium.mp. 44. vaginal maturation.mp. 45. vaginal maturation index.mp. 46. e2.mp. 47. exp estradiol/ 48. estradiol.mp. 49. oestradiol.mp. 50. exp estrogen/ 51. estrogen.mp. 52. oestrogen.mp. 53. 38 or 39 or 40 or 41 or 42 or 43 or 44 or 45 or 46 or 47 or 48 or 49 or 50 or 51 or 52 54. 37 and 53 55. randomized controlled trial'/ 56. controlled clinical trial'/ 57. random*'.ti,ab,tt. 58. randomization'/ 59. placebo.ti,ab,tt. 60. (compare or compared or comparison).ti,tt. 61. (evaluated or evaluate or evaluating or assessed or assess).ab. 62. (compare or compared or comparing or comparison).ab. 63. 61 and 62 64. (open adj label).ti,ab,tt. 65. ((double or single or doubly or singly) adj (blind or blinded or blindly)).ti,ab,tt. 66. double blind procedure/ 67. (parallel adj group*).ti,ab,tt. 68. (crossover or "cross over").ti,ab,tt. 69. ((assign* or match or matched or allocation) adj6 (alternate or group or groups or intervention or interventions or patient or patients or subject or subjects or participant or participants)).ti,ab,tt. 70. (assigned or allocated).ti,ab,tt. 71. (controlled adj8 (study or design or trial)).ti,ab,tt. 72. (volunteer or volunteers).ti,ab,tt. 73. human experiment/ 74. trial.ti,tt. 75. 55 or 56 or 57 or 58 or 59 or 60 or 63 or 64 or 65 or 66 or 67 or 68 or 69 or 70 or 71 or 72 or 73 or 74 76. (random* adj sampl* adj8 ("cross section*" or questionnaire* or survey or surveys or database or databases)).ti,ab,tt. not (comparative study/ or controlled study/ or "randomised controlled".ti,ab,tt. or "randomized controlled".ti,ab,tt. or "randomly assigned".ti,ab,tt.) 77. cross-sectional study/ not (randomized controlled trial/ or controlled clinical study/ or controlled study/ or "randomised controlled".ti,ab,tt. or "randomized controlled".ti,ab,tt. or "control group".ti,ab,tt. or "control groups".ti,ab,tt.) 78. (("case control*" and random*) not ("randomised controlled" or "randomized controlled")).ti,ab,tt. 79. ("systematic review" not (trial or study)).ti,tt. 80. (nonrandom* not random*).ti,ab,tt. 81. "random field*".ti,ab,tt. 82. ("random cluster" adj4 sampl*).ti,ab,tt. 83. (review.ab. and review.pt.) not trial.ti,tt. 84. "we searched".ab. and (review.ti,tt. or review.pt.) 85. "update review".ab. 86. (databases adj5 searched).ab. 87. (rat or rats or mouse or mice or swine or porcine or murine or sheep or lambs or pigs or piglets or rabbit or rabbits or cat or cats or dog or dogs or cattle or bovine or monkey or monkeys or trout or marmoset*).ti,tt. and animal experiment/ 88. animal experiment/ not (human experiment/ or human/) 89. 76 or 77 or 78 or 79 or 80 or 81 or 82 or 83 or 84 or 85 or 86 or 87 or 88 90. 75 not 89 91. 54 and 90 | 1. soy.mp. 2. isoflavon*.mp. 3. [exp Isoflavones/] 4. [exp Soybeans/] 5. soy protein.mp. 6. [exp Soybean Proteins/] 7. [exp Soy Foods/] 8. [exp Soy Milk/] 9. (soy adj3 milk).mp. 10. soybeans.mp. 11. (soy adj3 bean$).mp. 12. soy food$.mp. 13. (soy adj3 beverage$).mp. 14. isolated soy protein.mp. 15. soy protein isolate*.mp. 16. soya.mp. 17. phytoestrogens.mp. 18. Tofu*.mp. 19. Natto*.mp. 20. Tempeh*.mp. 21. Miso*.mp. 22. exp genistein/ 23. genistein.mp. 24. genistin.mp. 25. daidzein.mp. 26. exp daidzin/ 27. exp equol/ 28. equol.mp. 29. glycitein.mp. 30. glycitin.mp. 31. 1/30 OR 32. FSH.mp. 33. exp Follicle Stimulating Hormone/ 34. follicle stimulating hormone.mp. 35. endometrial thickness.mp. 36. endometrial.mp. 37. exp endometrium/ 38. endometrium.mp. 39. vaginal maturation.mp. 40. vaginal maturation index.mp. 41. E2.mp. 42. exp estradiol/ 43. estradiol.mp. 44. oestradiol.mp. 45. exp estrogen/ 46. estrogen.mp. 47. oestrogen.mp. 48. 32/47 OR 49. 31 AND 48 |

# **Supplementary Table 2.** PICOTS framework of the search strategy

| **PICOTS framework defined in the present systematic review and meta-analysis** | | | | | |
| --- | --- | --- | --- | --- | --- |
| **Participants** | **Interventions** | **Comparators** | **Outcomes** | **Time** | **Study design** |
| Post-menopausal women | Dietary interventions containing isoflavones from soy | Suitable non-isoflavone containing control | Endometrial thickness, vaginal maturation index, levels of follicle-stimulating hormone (FSH) and levels of estradiol, mean difference and 95% confidence intervals | ≥3 months | Randomized controlled trials in humans |

PICOTS, participants, interventions, comparators, outcomes, time and study design

# **Supplementary Table 3.** Table of characteristics of the randomized controlled trials assessing the effect of soy isoflavones on measures of estrogenicity


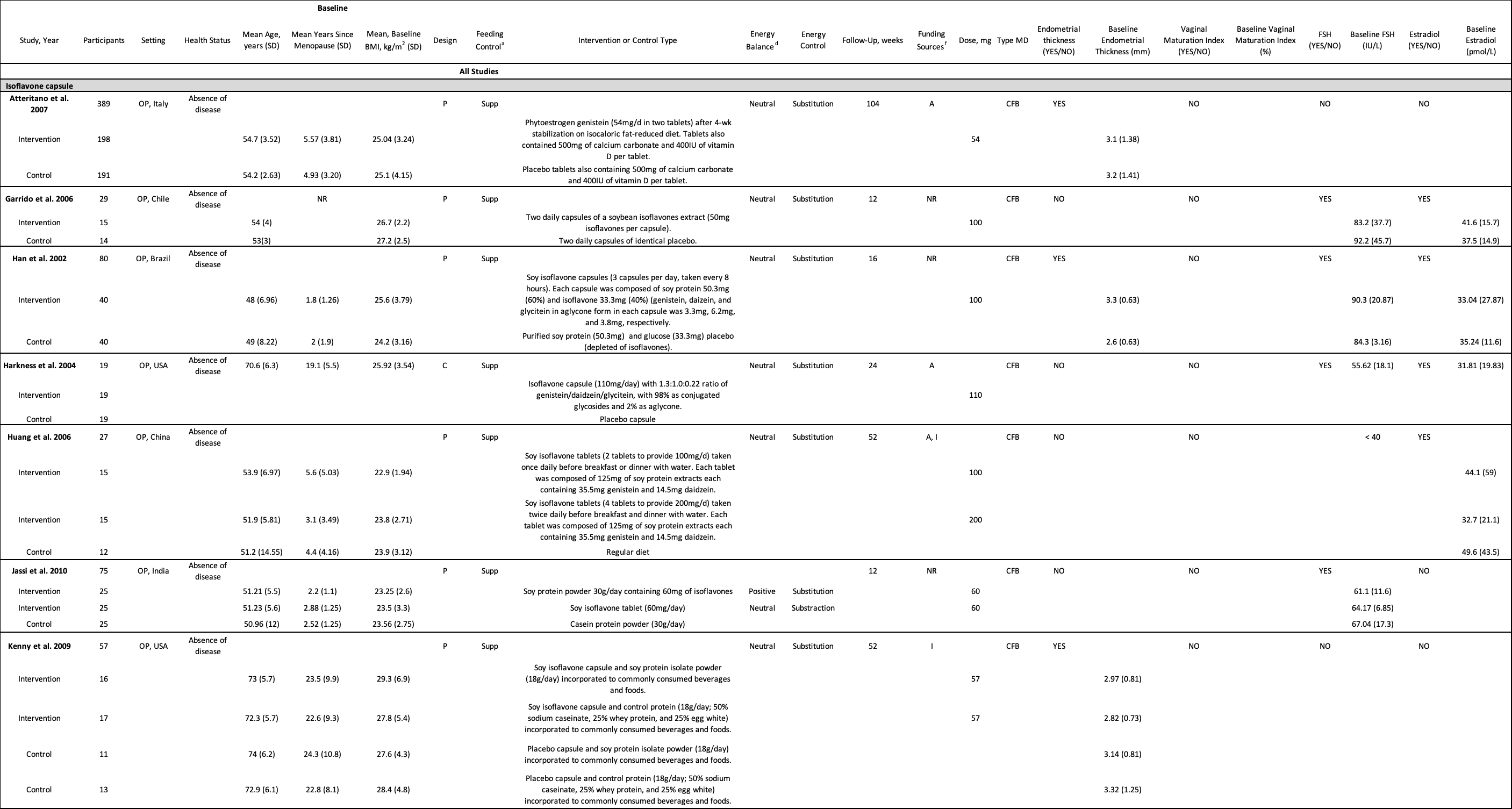


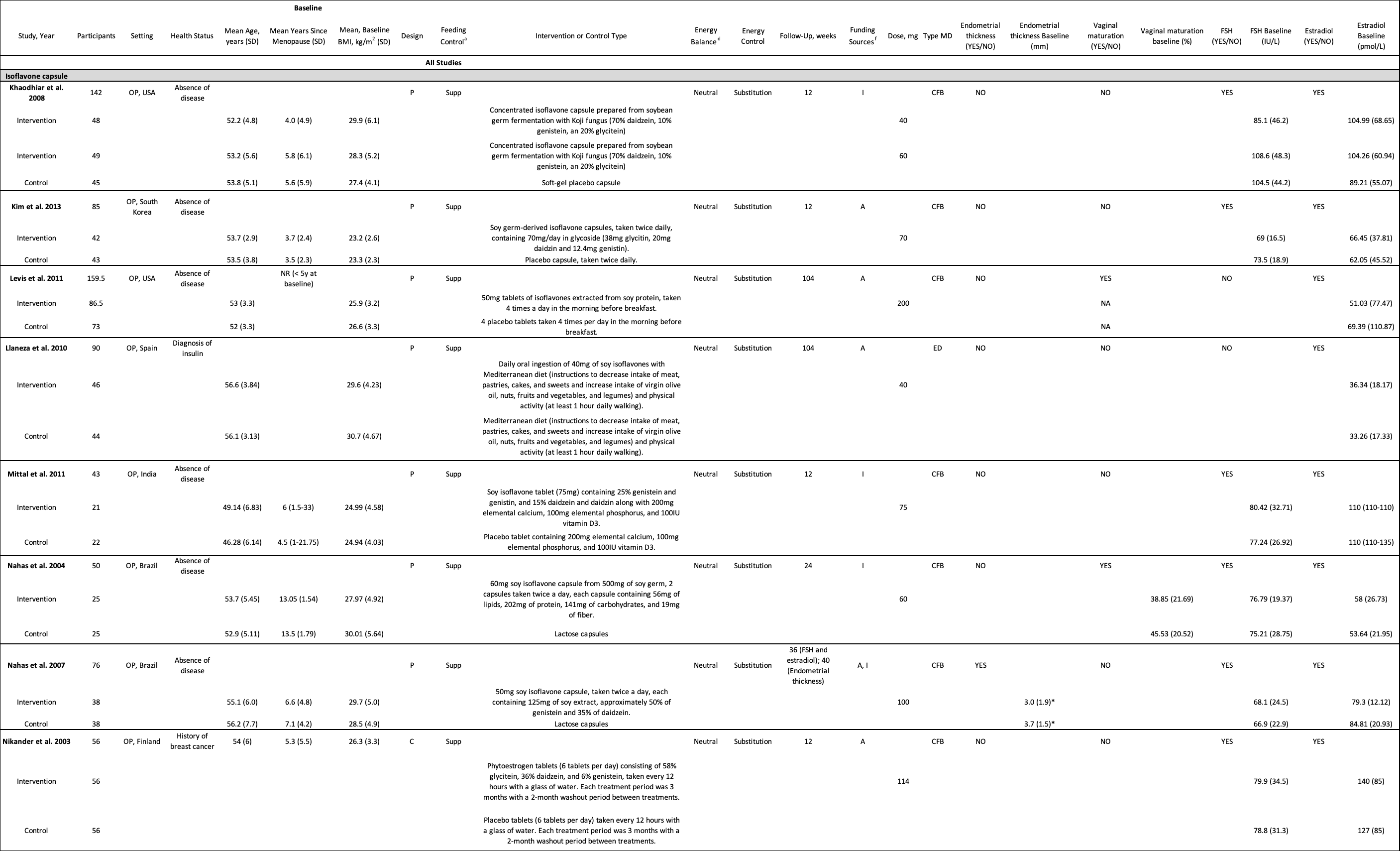


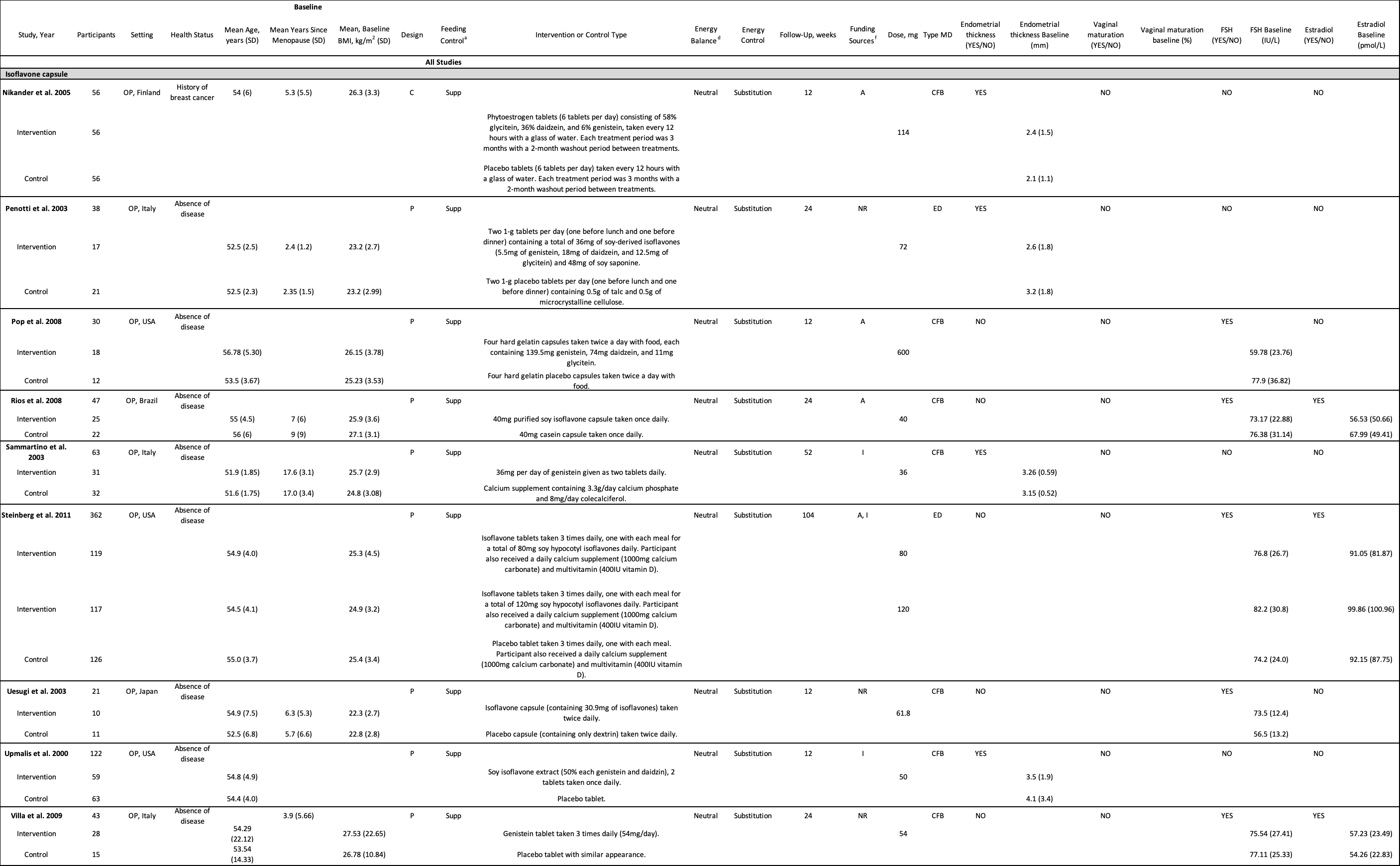


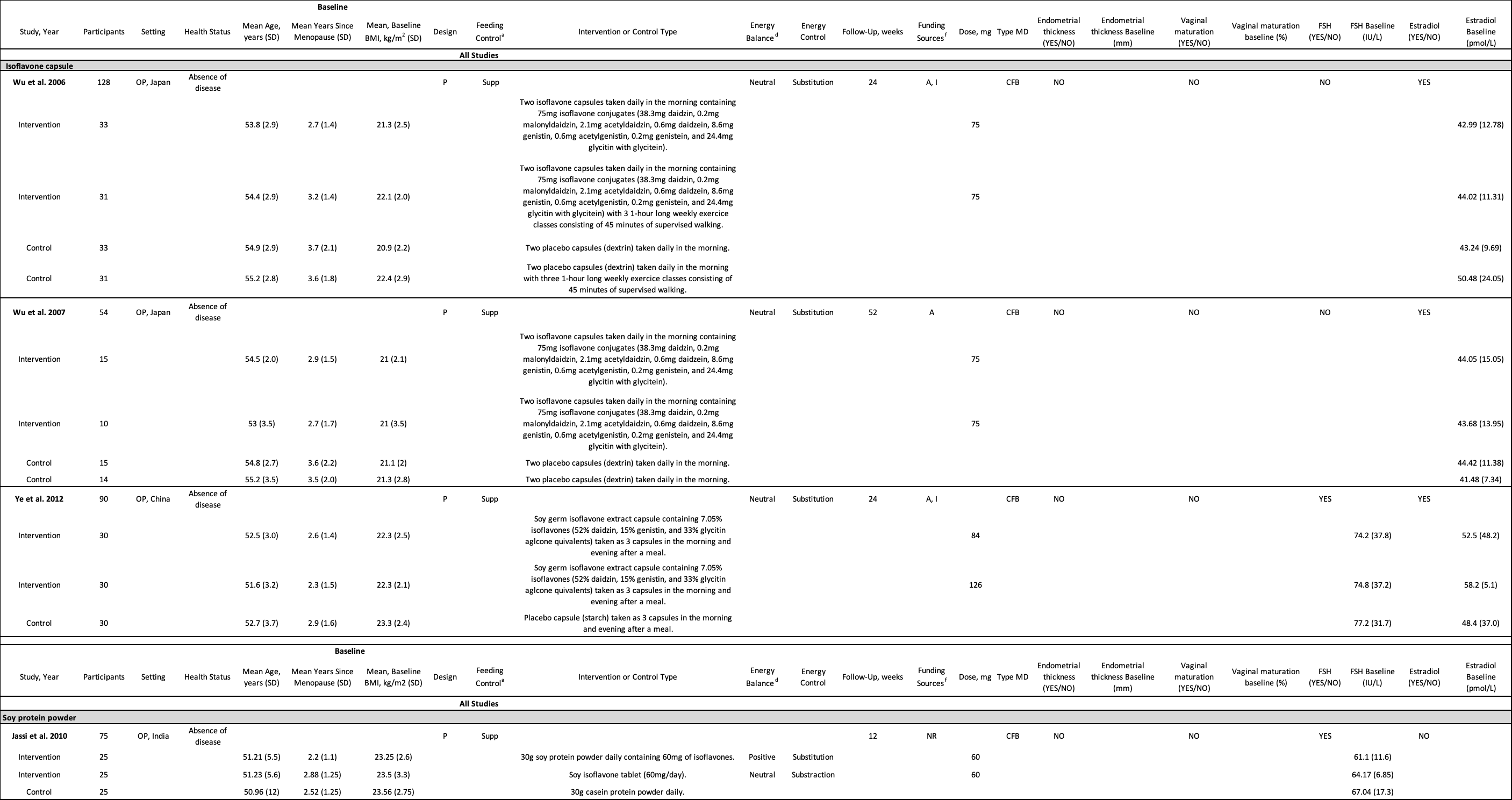


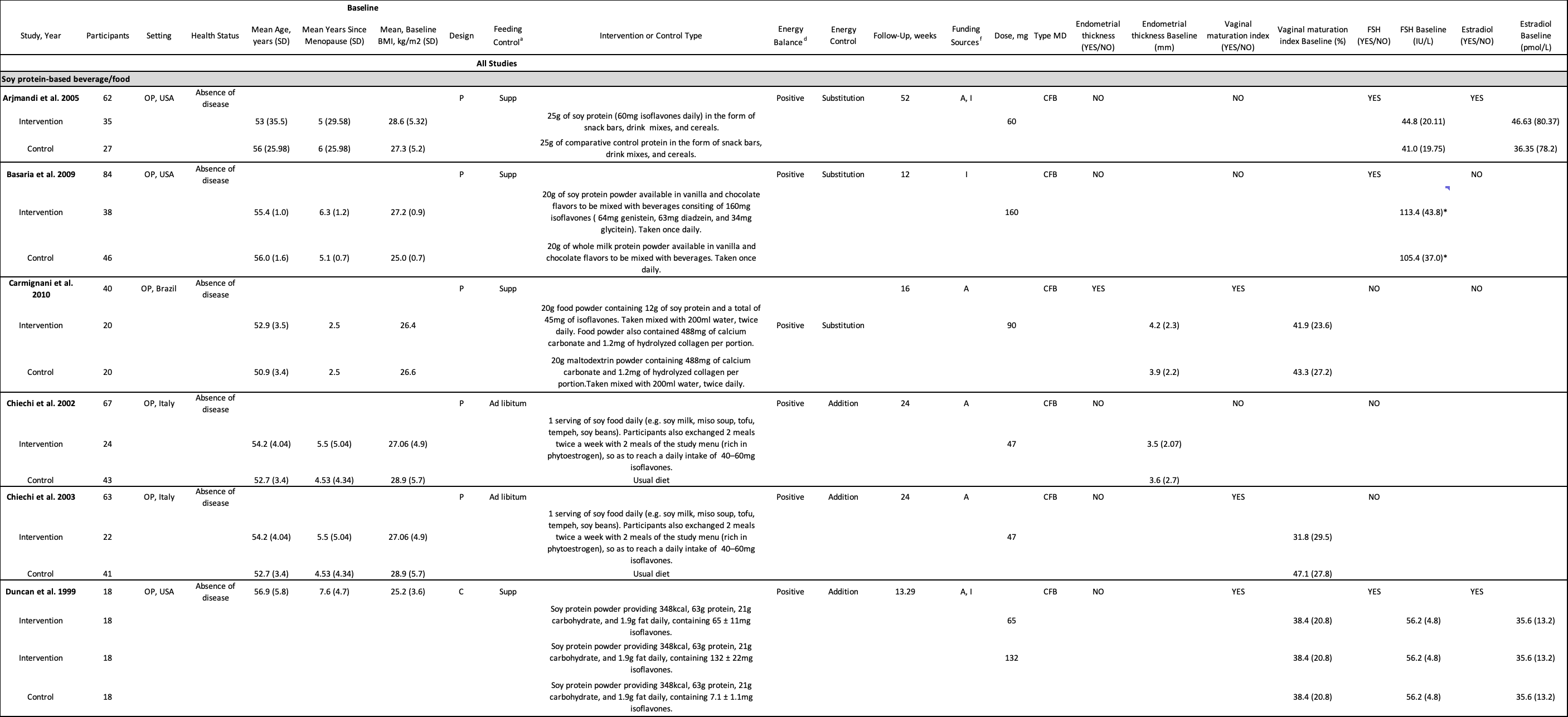


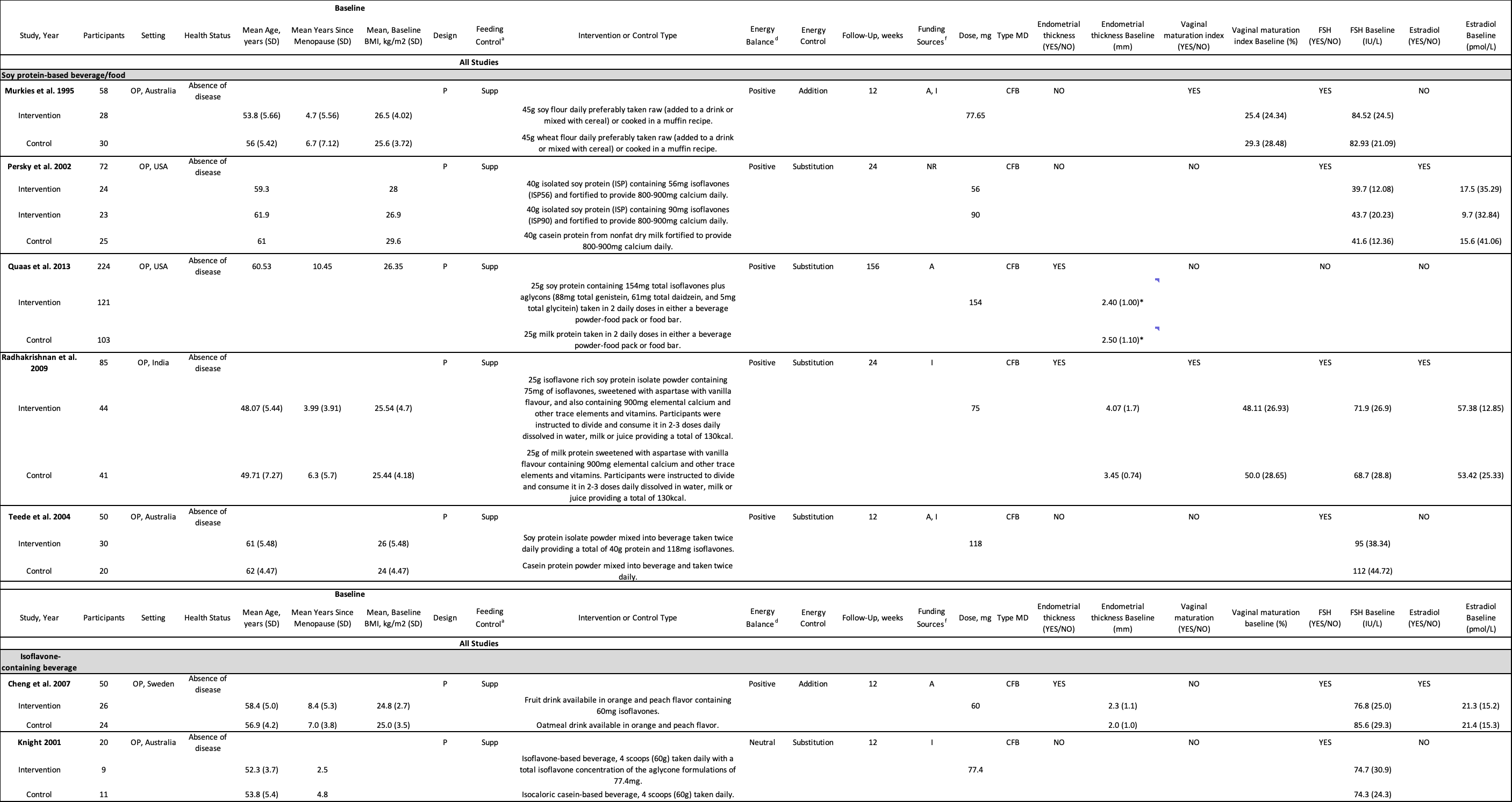


A, agency; BMI, body mass index; C, crossover; FSH, follicle-stimulating hormone; I, industry; NR, not reported; OP, outpatient; P, parallel; SD, standard deviation; Supp, supplemental feeding control; USA, United States of America

^a^ Supplemental feeding control (Supp) is the provision of some foods consumed during the study. Ad libitum feeding control refers to foods being available at all time.

^d^ Neutral energy balance refers to the maintenance of usual energy intake. Positive energy balance refers to a greater than normal energy intake. Negative energy balance refers to a deficit in normal energy intake.

^f^ Agency funding is that from government, university, or not-for-profit sources. Industry funding is that from trade organisations that obtain revenue from the sale of products.

*Median and interquartile range (IQR)

# **Supplementary Table 4.** Assessment of acceptability and adverse events*

| **Study** | **Assessment of Acceptability** | **Assessment of Adverse Events** |
| --- | --- | --- |
| **Isoflavone capsule** | | |
| **Atteritano et al. 2017** | NR | In the placebo group, 3 women withdrew due to abdominal pain, 3 for dyspepsia, 3 for vomiting and 6 for constipation. In the isoflavone group, 6 withdrew due to abdominal pain, 5 for epigastric pain, 9 for dyspepsia, 4 for vomiting, and 13 for constipation. |
| **Garrido et al. 2006** | NR | In the isoflavone group, 1 woman reported abdominal bloating and 1 reported nicturia. |
| **Han et al. 2002** |  | One participant withdrew due to "poor response" and 1 due to nausea in the isoflavone and placebo group respectively. None of the women experienced abnormal bleeding or side effects after the study. |
| **Jassi et al. 2010** | NR | 20% of women consuming soy protein and soy isoflavones experienced GI symptoms including bloating, abdominal pain, and constipation, with incidental occurrences of nausea and vomiting in 1 woman each within both groups. |
| **Kenny et al. 2009** | NR | The adverse events rate did not differ between isoflavone and control groups. Women experienced gastrointestinal disturbances (n=10) and differences in mammogram or breast tenderness (n=2). |
| **Khaodhiar et al. 2008** | NR | The isoflavone groups' primary side effects were GI, including stomach upset, constipation, flatulence, and diarrhea, causing 2 participants to discontinue the 60 mg isoflavone supplement (due to stomach upset and diarrhea respectively). |
| **Levis et al. 2011** | NR | Nearly all participants experienced adverse events, evenly distributed between both groups, with a slightly higher percentage of participants consuming soy isoflavones reporting constipation compared to those on placebo (31.2% vs. 20.6%). Vaginal bleeding was reported by 13.9% of women in the soy isoflavone group and 14.3% in the placebo group. |
| **Mittal et al. 2011** | NR | At 4-week, 1 woman from the isoflavone group discontinued treatment due to sleep disturbances, anxiety, and restlessness. In the placebo group, 2 women encountered generalized body aches and increased fatigue, leading to discontinuation at 2 weeks and 2 days respectively. The occurrence of adverse events was comparable between both groups. |
| **Nahas et al. 2004** | NR | In the isoflavone group, 3 women reported constipation, 2 reported flatulence and 2 reported nausea, while in the placebo group, 2 reported constipation and flatulence. |
| **Nahas et al. 2007** | NR | 7 women in the isoflavone group (18.4%) and 4 in the placebo group (10.5%) reported gastrointestinal-related adverse events. |
| **Nikander et al. 2003** | NR | During the soy isoflavone phase, 2 women withdrew from the study because of stomachache. During the control phase, 2 women withdrew due to vaginal bleeding and lack of effect respectively. |
| **Penotti et al. 2003** | NR | 1 woman in the soy isoflavone group withdrew due to diarrhea onset. |
| **Pop et al. 2008** | NR | In the soy isoflavone group, 1 woman reported nausea, 6 reported increased hot flashes, and 2 reported increased breast size/tenderness. In the placebo group, 1 woman reported flatulence, 1 reported swelling in legs, 1 reported breast tenderness, 3 reported increased hot flashes, and 1 reported decreased hot flashes intensity. |
| **Soy protein-based beverage/foods** | | |
| **Arjmandi et al. 2005** | In the control group, women withdrew from the study for dislike of the volume or flavor of food (n=3). | In the control group, women withdrew from the study due to gastrointestinal side effects (n=2), and headaches caused by study food (n=1). |
| **Basaria et al. 2009** | 2 women withdrew from the study due to bad taste of the study foods. | 5 women withdrew from the study due to gastrointestinal upset. |
| **Carmignani et al. 2010** | NR | In the soy isoflavone group, 2 women reported mastalgia, 1 reported bleeding, 1 reported headache, 3 reported water retention and 1 reported intestinal complaints. In the control group, 1 woman reported bleeding, 3 reported nausea, 1 reported water retention and 1 reported intestinal complaints. There were no statistical differences in reported adverse events between groups. |
| **Chienchi et al. 2002** | In the diet group, women withdrew from the study due to dislike of soy (n=11), study being "too difficult" (n=7), foods being difficult to find (n=3) and high cost of the foods (n=3). | NR |
| **Radhakrishnan et al. 2009** | Women reported equal acceptability to each soy protein and milk protein preparations (acceptability score P=0.540). The amount of powder instructed was difficult to follow. | 2 women in the isoflavone group and 3 women in the control group withdrew from the trial due to gastrointestinal side effects and food intolerance (constipation, bloating, nausea, and vomiting). |
| **Isoflavone-containing beverages** | | |
| **Knight 2001** | 5 women in the isoflavone group and 1 in the control group reported dislike of taste (P=0.07). | Rates of adverse events was significantly higher in the isoflavone group compared to control group (P<0.001) and included bloating (n=4), nausea (n=3), and changes in bowel function (n=2). |

*Of the 40 trials, 5 reported some assessment of acceptability and 18 reported some assessment of adverse events. Trials not listed in the table did not report on acceptability and adverse events.

# **Supplementary Table 5.** Sensitivity analyses of the use of correlation coefficients of 0.25 and 0.75 for crossover trials in the primary analysis of the effect of soy isoflavones on measures of estrogenicity

|  | **MD (95% CI), P-value**  **I^2^, P-value** | | |
| --- | --- | --- | --- |
|  | **Correlation Coefficient**  **used in the Primary Analysis** | **Correlation Coefficient used in**  **Sensitivity Analyses** | |
| **Outcomes (no. crossover trial comparisons/total)** | **0.5** | **0.25** | **0.75** |
| Endometrial thickness (1/14) | -0.22 [-0.45, 0.01], P_MD_=0.059  I^2^=69.3%, P_Q_<0.001 | -0.22 [-0.45, 0.01], P_MD_=0.062  I^2^=67.3%, P_Q_<0.001 | -2.23 [-0.47, 0.01], P_MD_=0.060  I^2^=73.6%, P_Q_<0.001 |
| Vaginal maturation index (2/8) | 2.31 [-2.14, 6.75], P_MD_=0.310  I^2^=1.3%, P_Q_=0.420 | 2.96 [-1.62, 7.55], P_MD_=0.205  I^2^=0.0%, P_Q_=0.534 | 1.24 [-3.58, 6.07], P_MD_=0.614  I^2^=25.8%, P_Q_=0.223 |
| Follicle-stimulating hormone (4/31) | -0.02 [-2.39, 2.35], P_MD_=0.987  I^2^=51.9%, P_Q_<0.001 | -0.03 [-2.51, 2.46], P_MD_=0.984  I^2^=51.6%, P_Q_=0.001 | -0.00 [-2.15, 2.15], P_MD_=0.999  I^2^=52.6%, P_Q_<0.001 |
| Estradiol (4/31) | 1.61 [-1.17, 4.38], P_MD_=0.256  I^2^=23.5%, P_Q_=0.121 | 1.65 [-1.14, 4.43], P_MD_=0.246  I^2^=20.7, P_Q_=0.154 | 1.54 [-1.23, 4.31], P_MD_=0.276  I^2^=29.9, P_Q_=0.061 |

CI, confidence interval; MD, mean difference

# **Supplementary Table 6.** GRADE assessment of study quality

| **Outcome and trial (N)** | **Design** |  | **GRADE assessment** | | | | | |  | |  |  |
| --- | --- | --- | --- | --- | --- | --- | --- | --- | --- | --- | --- | --- |
|  |  | **Downgrades** | | | | |  | **Upgrades** |  | |  |  |
|  |  | **ROB** | **Inconsistency** | **Indirectness** | **Imprecision** | **Publication bias** |  | **Dose response** | **Effect (MD [95% CI], P_MD_)** | | **Certainty of Evidence^a^** | **Interpretation of magnitude of effect^b^** |
| Endometrial thickness (14) | Randomized trials | Not serious | Serious^1^ | Not serious | Not serious^2^ | None |  | None | ↔ | -0.22 mm [-0.45 to 0.01], P=0.059 | ⨁⨁⨁◯ Moderate | No effect |
| Vaginal maturation index (8) | Randomized trials | Not serious | Not serious | Not serious | Serious^3^ | None^4^ |  | None | ↔ | 2.31 [-2.14 to 6.75],  P=0.310 | ⨁⨁⨁◯ Moderate | No effect |
| Follicle-  stimulating hormone (31) | Randomized trials | Not serious | Not serious^5^ | Not serious | Not serious | None |  | None | ↔ | 0.02 IU/L [-2.39 to 2.35], P=0.987 | ⨁⨁⨁⨁ High | No effect |
| Estradiol (31) | Randomized trials | Not serious | Not serious | Not serious | Not serious | None |  | None | ↔ | 1.61 pmol/L [-1.17 to 4.38], P=0.256 | ⨁⨁⨁⨁ High | No effect |

^a^ Since all included trials were randomized controlled trials, the certainty of the evidence was graded as high for all outcomes by default and then downgraded or upgraded based on pre-specified criteria. Criteria for downgrades included risk of bias (ROB) (downgraded if the majority of trials were considered to be at high ROB); inconsistency (downgraded if there was substantial unexplained heterogeneity [I^2^ ≥ 50%, P_Q_ < 0.10]; indirectness (downgraded if there were factors absent or present relating to the participants, interventions, or outcomes that limited the generalizability of the results); imprecision (downgraded if the 95% confidence interval crossed the minimally important difference [MID] for harm set at 0.30 for endometrial thickness, 3.8 for vaginal maturation index, 7.4 for FSH and 5.2 for estradiol (10% of baseline mean of 3.0mm, 37.6, 7.4IU/L, and 5.2pmol/L, respectively); and publication bias (downgraded if there is evidence of publication bias based on funnel plot asymmetry and/or significant Egger’s or Begg’s tests (P<0.10) with confirmation by adjustment by Duval and Tweedie trim-and-fill analysis). Criteria for upgrades included a significant dose-response gradient.

^b^ For the interpretation of the magnitude, we used the MIDs (see a above) to assess the importance of magnitude of our pooled estimates using the effect size categories according to new GRADE guidance. We then used the MIDs to assess the importance of the magnitude of our point estimates using the effect size categories according GRADE guidance (Santesso et al. 2020, Schunemann et al. 2013, Balshem et al. 2011) as follows: large effect (≥5x MID); moderate effect (≥2x MID); small important effect (≥1x MID); and trivial/unimportant effect (<1 MID).

CI, confidence interval; MD, mean difference; ROB, risk of bias

^1^ Downgrade for serious inconsistency, due to substantial unexplained heterogeneity I^2^=69.3%, P_Q_ <0.001.

^2^ No downgrade for imprecision. Although in sensitivity analyses removal of 4 individual trials resulted in a gain of significance for a reduction in endometrial thickness, we did not downgrade for imprecision as we were assessing evidence for estrogenicity.

^3^ Downgrade for serious imprecision as the 95% confidence interval overlaps the MID of clinically important change (3.8, 10% of baseline) for vaginal maturation index reflecting potential estrogenicity.

^4^ No downgrade for publication bias, as publication bias could not be assessed due to lack of power for assessing funnel plot asymmetry and small study effects (<10 trial comparisons included in the meta-analysis).

^5^ No downgrade for serious inconsistency. Although there was substantial heterogeneity in the analysis of the effect of isoflavones from soy on FSH, we did not downgrade for serious inconsistency, since it was partially explained when the study by Jassi et al. 2010 (powder) or (tablet) or Kim et al. 2013 was removed as part of a priori sensitivity analyses (Original: I^2^=52%, P_Q_<0.001; after study removed: I^2^=39% P_Q_=0.015, 37% P_Q_=0.025, 47%, P_Q_=0.002, respectively).

# **Supplementary Table 7.** Effects of hormone replacement therapy on estrogen-related intermediate outcomes compared to soy isoflavones

|  | **Endometrial thickness** | **Vaginal maturation index** | **Follicle-stimulating hormone** | **Estradiol** |
| --- | --- | --- | --- | --- |
| **Systematic reviews and meta-analyses^1,2^** | | | | |
| HRT | ↑^*^ | ↑ | ↓^**^ | ↑^**^ |
| Soy isoflavones | ↔ | ↔ | ↔ | ↔ |
| **Head-to-head trial^3^** | | | | |
| HRT | ↔ | ↑^***^ | ↓^†^ | ↑^†^ |
| Soy isoflavones | ↔ | ↔ | ↔ | ↔ |

^1^Furness S, Roberts H, Marjoribanks J, Lethaby A. Hormone therapy in postmenopausal women and risk of endometrial hyperplasia. Cochrane Database Syst Rev. 2012 Aug 15;2012(8):CD000402. doi: 10.1002/14651858.CD000402.pub4. PMID: 22895916; PMCID: PMC7039145.

^2^Lu, D. H., Zhou, S. Y., & Xu, L. Z. (2023). Association between hormone replacement therapy and sex hormones in postmenopausal women: a systematic review and meta-analysis. European review for medical and pharmacological sciences, 27(11), 5264–5279. https://doi.org/10.26355/eurrev_202306_32646

^3^Carmignani, L. O., Pedro, A. O., Costa-Paiva, L. H., & Pinto-Neto, A. M. (2010). The effect of dietary soy supplementation compared to estrogen and placebo on menopausal symptoms: a randomized controlled trial. Maturitas, 67(3), 262–269. https://doi.org/10.1016/j.maturitas.2010.07.007

^*^Statistically significantly increased risk of endometrial hyperplasia at two and three years, across different dosage levels

^**^ Statistically significantly decrease in follicle-stimulating hormone and increase in estradiol after 3 months for both oral and transdermal treatments

^***^Statistically significant increase of 18.1 in mean vaginal maturation value after 16 weeks of treatment

^†^Statistically significant 46.2% decrease and 513.7% increase in mean percentage variation of follicle-stimulating hormone and estradiol respectively after 16 weeks of treatment

# **Supplementary Figure 1.** Risk of bias proportion graph for the effect of soy isoflavones on measures of estrogenicity in parallel trials

**
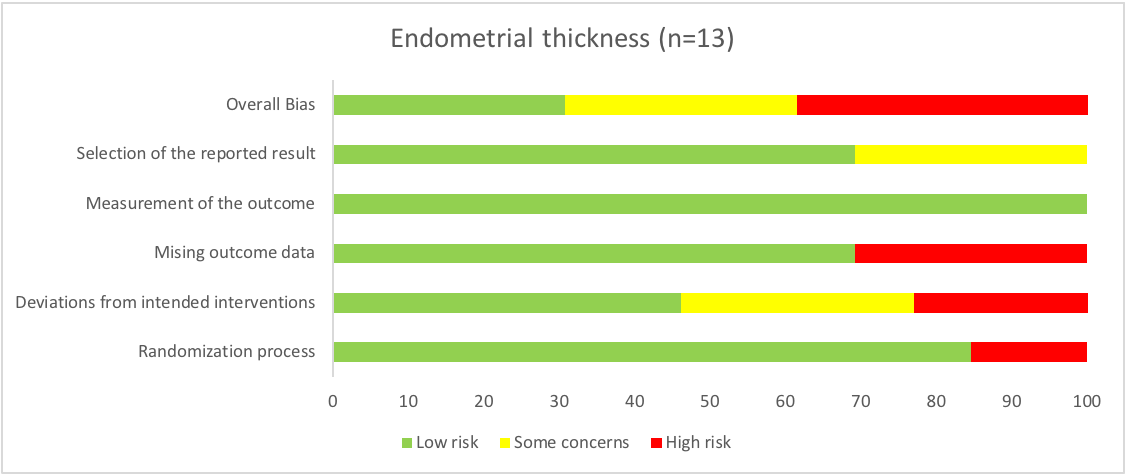
**

**
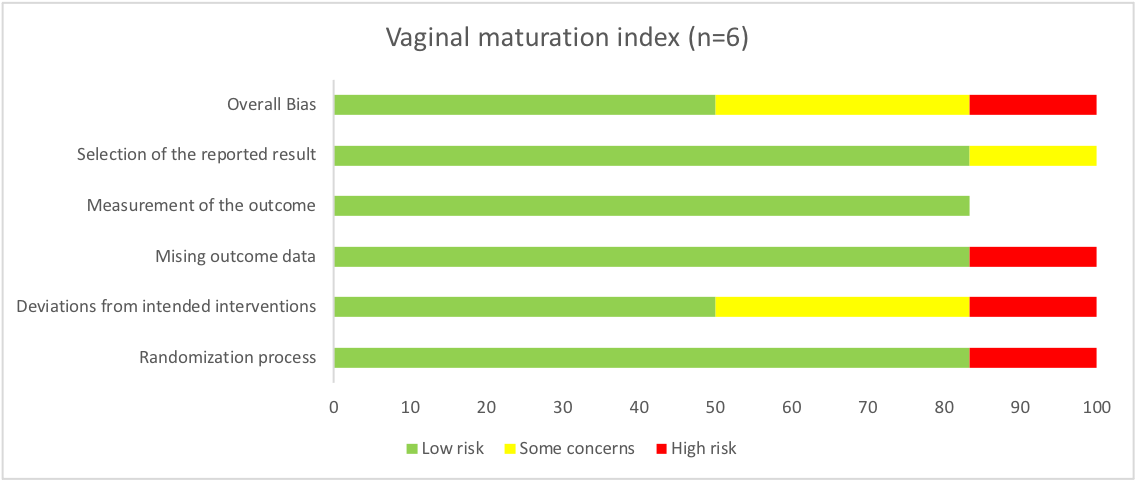
**

**
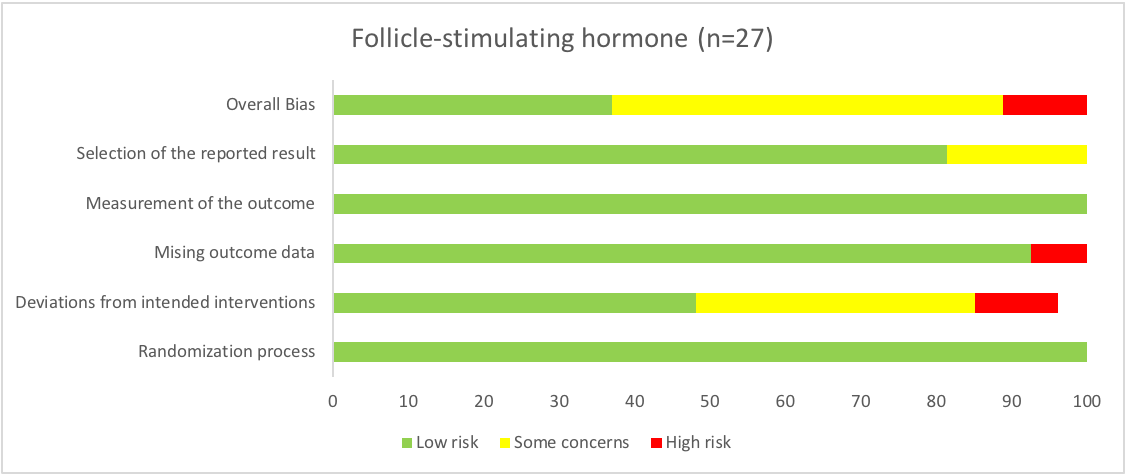
**

**
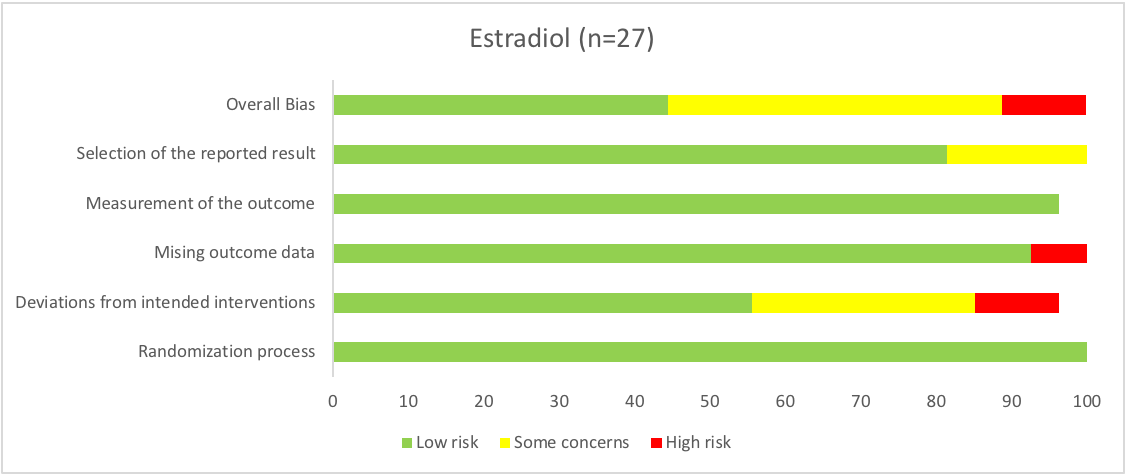
**

# **Supplementary Figure 2.** Risk of bias proportion graph for the effect of soy isoflavones on measures of estrogenicity in crossover trials


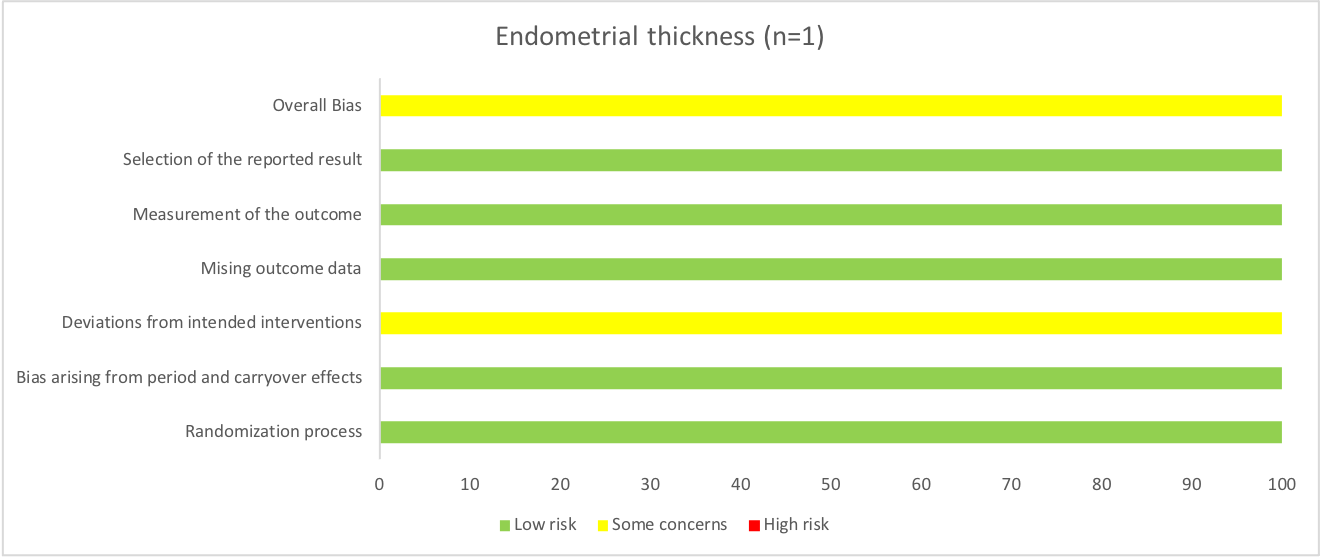


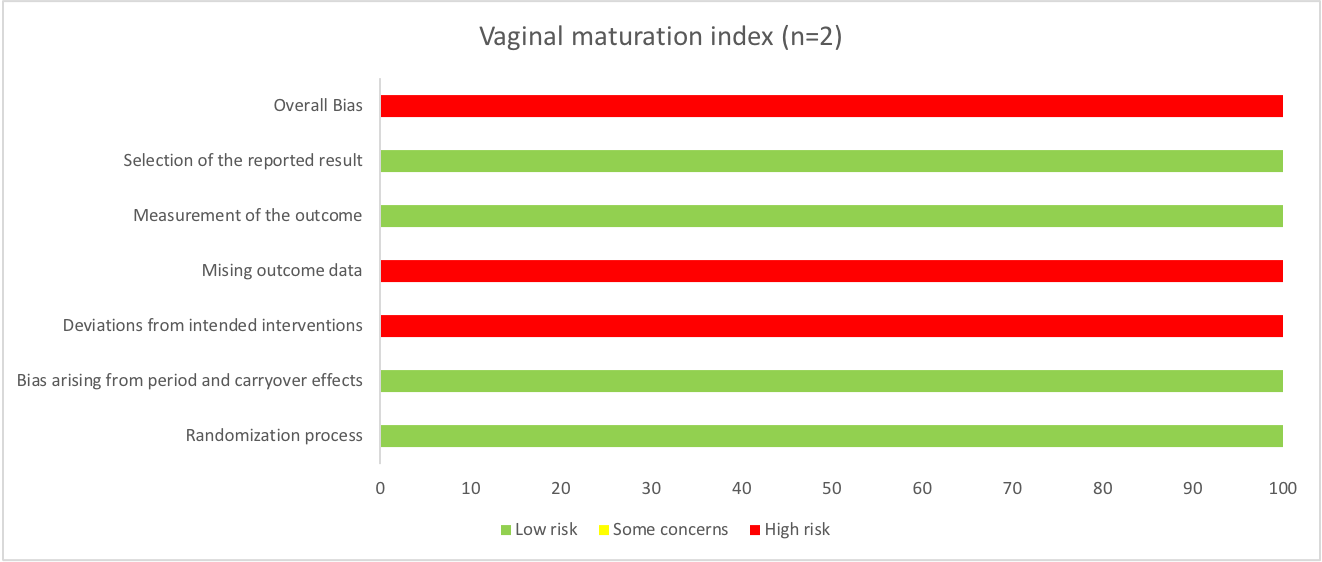


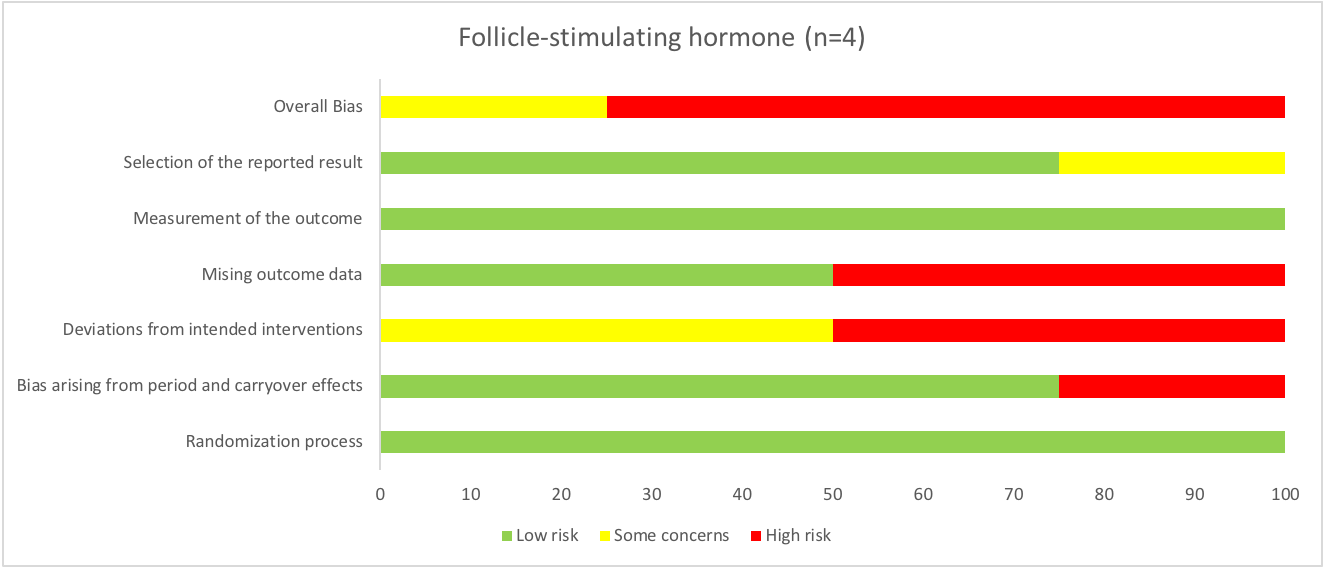


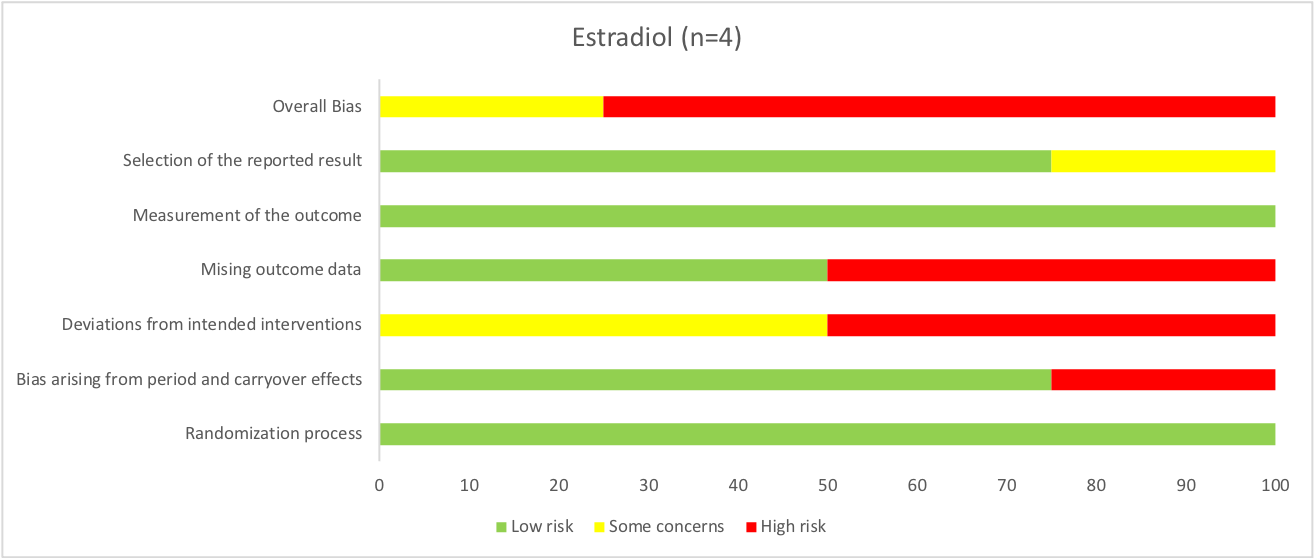


# **Supplementary Figure 3.** Forest plot of randomized controlled trials of the effect of soy isoflavones on endometrial thickness

Pooled effect estimates for each subgroup and overall effect are represented by the diamonds. Data are expressed as weighted mean differences with 95% confidence intervals using the generic inverse-variance method and random effects DerSimonian-Laird model. Paired analyses were applied to all crossover trials. Inter‐study heterogeneity was assessed using the Cochrane Q statistic and quantified using the I^2^ statistic, with significance set at p<0.100 and I^2^≥50% considered to be evidence of substantial heterogeneity.

Risk of Bias Legend: (H) High Risk; (L) Low Risk; (S) Some concerns. The letters represent the following risk of bias domains: A, randomization process; B, bias arising from period and carryover effects; C, deviations from intended intervention; D, missing outcome data; E, measurement of the outcome; F, selection of the reported result; and G, overall bias. Risk of bias arising from period and carryover effects was only applicable to crossover trials.

The pooled effect summary was calculated with the χ^2^ test. The test for group differences was calculated with meta-regression, which uses the Wald test.

CI, confidence interval

# **Supplementary Figure 4.** Forest plot of randomized controlled trials of the effect of soy isoflavones on vaginal maturation index

Pooled effect estimates for each subgroup and overall effect are represented by the diamonds. Data are expressed as weighted mean differences with 95% confidence intervals using the generic inverse-variance method and random effects DerSimonian-Laird model. Paired analyses were applied to all crossover trials. Inter‐study heterogeneity was assessed using the Cochrane Q statistic and quantified using the I^2^ statistic, with significance set at p<0.100 and I^2^≥50% considered to be evidence of substantial heterogeneity.

Risk of Bias Legend: (H) High Risk; (L) Low Risk; (S) Some concerns. The letters represent the following risk of bias domains: A, randomization process; B, bias arising from period and carryover effects; C, deviations from intended intervention; D, missing outcome data; E, measurement of the outcome; F, selection of the reported result; and G, overall bias. Risk of bias arising from period and carryover effects was only applicable to crossover trials.

The pooled effect summary was calculated with the χ^2^ test. The test for group differences was calculated with meta-regression, which uses the Wald test.

CI, confidence interval

# **Supplementary Figure 5.** Forest plot of randomized controlled trials of the effect of soy isoflavones on follicle-stimulating hormone

Pooled effect estimates for each subgroup and overall effect are represented by the diamonds. Data are expressed as weighted mean differences with 95% confidence intervals using the generic inverse-variance method and random effects DerSimonian-Laird model. Paired analyses were applied to all crossover trials. Inter‐study heterogeneity was assessed using the Cochrane Q statistic and quantified using the I^2^ statistic, with significance set at p<0.100 and I^2^≥50% considered to be evidence of substantial heterogeneity.

Risk of Bias Legend: (H) High Risk; (L) Low Risk; (S) Some concerns. The letters represent the following risk of bias domains: A, randomization process; B, bias arising from period and carryover effects; C, deviations from intended intervention; D, missing outcome data; E, measurement of the outcome; F, selection of the reported result; and G, overall bias. Risk of bias arising from period and carryover effects was only applicable to crossover trials.

The pooled effect summary was calculated with the χ^2^ test. The test for group differences was calculated with meta-regression, which uses the Wald test.

CI, confidence interval

# **Supplementary Figure 6.** Forest plot of randomized controlled trials of the effect of soy isoflavones on estradiol

Pooled effect estimates for each subgroup and overall effect are represented by the diamonds. Data are expressed as weighted mean differences with 95% confidence intervals using the generic inverse-variance method and random effects DerSimonian-Laird model. Paired analyses were applied to all crossover trials. Inter‐study heterogeneity was assessed using the Cochrane Q statistic and quantified using the I^2^ statistic, with significance set at p<0.100 and I^2^≥50% considered to be evidence of substantial heterogeneity.

Risk of Bias Legend: (H) High Risk; (L) Low Risk; (S) Some concerns. The letters represent the following risk of bias domains: A, randomization process; B, bias arising from period and carryover effects; C, deviations from intended intervention; D, missing outcome data; E, measurement of the outcome; F, selection of the reported result; and G, overall bias. Risk of bias arising from period and carryover effects was only applicable to crossover trials.

The pooled effect summary was calculated with the χ^2^ test. The test for group differences was calculated with meta-regression, which uses the Wald test.

CI, confidence interval

# **Supplementary Figure 7.** Sensitivity analysis of the systematic removal of each trial for the effect of soy isoflavones on endometrial thickness

CI, confidence interval

# **Supplementary Figure 8.** Sensitivity analysis of the systematic removal of each trial for the effect of soy isoflavones on vaginal maturation index

CI, confidence interval

# **Supplementary Figure 9.** Sensitivity analysis of the systematic removal of each trial for the effect of soy isoflavones on follicle-stimulating hormone

CI, confidence interval

# **Supplementary Figure 10.** Sensitivity analysis of the systematic removal of each trial for the effect of soy isoflavones on estradiol

CI, confidence interval

# **Supplementary Figure 11.** Sensitivity analyses with the use of fixed effects models for the effect of soy isoflavones on endometrial thickness

Pooled effect estimates for each subgroup and overall effect are represented by the diamonds. Data are expressed as weighted mean differences with 95% confidence intervals using the generic inverse-variance method and random effects DerSimonian-Laird model. Paired analyses were applied to all crossover trials. Inter‐study heterogeneity was assessed using the Cochrane Q statistic and quantified using the I^2^ statistic, with significance set at p<0.100 and I^2^≥50% considered to be evidence of substantial heterogeneity.

Risk of Bias Legend: (H) High Risk; (L) Low Risk; (S) Some concerns. The letters represent the following risk of bias domains: A, randomization process; B, bias arising from period and carryover effects; C, deviations from intended intervention; D, missing outcome data; E, measurement of the outcome; F, selection of the reported result; and G, overall bias. Risk of bias arising from period and carryover effects was only applicable to crossover trials.

The pooled effect summary was calculated with the χ^2^ test. The test for group differences was calculated with meta-regression, which uses the Wald test.

CI, confidence interval

# **Supplementary Figure 12.** Sensitivity analyses with the use of fixed effects models for the effect of soy isoflavones on vaginal maturation index

Pooled effect estimates for each subgroup and overall effect are represented by the diamonds. Data are expressed as weighted mean differences with 95% confidence intervals using the generic inverse-variance method and random effects DerSimonian-Laird model. Paired analyses were applied to all crossover trials. Inter‐study heterogeneity was assessed using the Cochrane Q statistic and quantified using the I^2^ statistic, with significance set at p<0.100 and I^2^≥50% considered to be evidence of substantial heterogeneity.

Risk of Bias Legend: (H) High Risk; (L) Low Risk; (S) Some concerns. The letters represent the following risk of bias domains: A, randomization process; B, bias arising from period and carryover effects; C, deviations from intended intervention; D, missing outcome data; E, measurement of the outcome; F, selection of the reported result; and G, overall bias. Risk of bias arising from period and carryover effects was only applicable to crossover trials.

The pooled effect summary was calculated with the χ^2^ test. The test for group differences was calculated with meta-regression, which uses the Wald test.

CI, confidence interval

# **Supplementary Figure 13.** Sensitivity analyses with the use of fixed effects models for the effect of soy isoflavones on follicle-stimulating hormone

Pooled effect estimates for each subgroup and overall effect are represented by the diamonds. Data are expressed as weighted mean differences with 95% confidence intervals using the generic inverse-variance method and random effects DerSimonian-Laird model. Paired analyses were applied to all crossover trials. Inter‐study heterogeneity was assessed using the Cochrane Q statistic and quantified using the I^2^ statistic, with significance set at p<0.100 and I^2^≥50% considered to be evidence of substantial heterogeneity.

Risk of Bias Legend: (H) High Risk; (L) Low Risk; (S) Some concerns. The letters represent the following risk of bias domains: A, randomization process; B, bias arising from period and carryover effects; C, deviations from intended intervention; D, missing outcome data; E, measurement of the outcome; F, selection of the reported result; and G, overall bias. Risk of bias arising from period and carryover effects was only applicable to crossover trials.

The pooled effect summary was calculated with the χ^2^ test. The test for group differences was calculated with meta-regression, which uses the Wald test.

CI, confidence interval

# **Supplementary Figure 14.** Sensitivity analyses with the use of fixed effects models for the effect of soy isoflavones on estradiol

Pooled effect estimates for each subgroup and overall effect are represented by the diamonds. Data are expressed as weighted mean differences with 95% confidence intervals using the generic inverse-variance method and random effects DerSimonian-Laird model. Paired analyses were applied to all crossover trials. Inter‐study heterogeneity was assessed using the Cochrane Q statistic and quantified using the I^2^ statistic, with significance set at p<0.100 and I^2^≥50% considered to be evidence of substantial heterogeneity.

Risk of Bias Legend: (H) High Risk; (L) Low Risk; (S) Some concerns. The letters represent the following risk of bias domains: A, randomization process; B, bias arising from period and carryover effects; C, deviations from intended intervention; D, missing outcome data; E, measurement of the outcome; F, selection of the reported result; and G, overall bias. Risk of bias arising from period and carryover effects was only applicable to crossover trials.

The pooled effect summary was calculated with the χ^2^ test. The test for group differences was calculated with meta-regression, which uses the Wald test.

CI, confidence interval

# **Supplementary Figure 15 (1 of 3).** Subgroup analyses for the effect of soy isoflavones on endometrial thickness*

Pooled effect estimates for each subgroup and overall effect are represented by the diamonds. Data are expressed as weighted mean differences with 95% confidence intervals using the generic inverse-variance method and random effects DerSimonian-Laird model. Paired analyses were applied to all crossover trials. Inter‐study heterogeneity was assessed using the Cochrane Q statistic and quantified using the I^2^ statistic, with significance set at p<0.100 and I^2^≥50% considered to be evidence of substantial heterogeneity.

Risk of Bias Legend: (H) High Risk; (L) Low Risk; (S) Some concerns. The letters represent the following risk of bias domains: A, randomization process; B, bias arising from period and carryover effects; C, deviations from intended intervention; D, missing outcome data; E, measurement of the outcome; F, selection of the reported result; and G, overall bias. Risk of bias arising from period and carryover effects was only applicable to crossover trials.

The pooled effect summary was calculated with the χ^2^ test. The test for group differences was calculated with meta-regression, which uses the Wald test.

†Post-hoc subgroup analysis by continent

*N=2 trial comparisons missing data for age, and N=3 trial comparisons missing data for years since menopause

CI, confidence interval: MD, mean difference

# **Supplementary Figure 15 (2 of 3).** Subgroup analyses for the effect of soy isoflavones on endometrial thickness*

Pooled effect estimates for each subgroup and overall effect are represented by the diamonds. Data are expressed as weighted mean differences with 95% confidence intervals using the generic inverse-variance method and random effects DerSimonian-Laird model. Paired analyses were applied to all crossover trials. Inter‐study heterogeneity was assessed using the Cochrane Q statistic and quantified using the I^2^ statistic, with significance set at p<0.100 and I^2^≥50% considered to be evidence of substantial heterogeneity.

Risk of Bias Legend: (H) High Risk; (L) Low Risk; (S) Some concerns. The letters represent the following risk of bias domains: A, randomization process; B, bias arising from period and carryover effects; C, deviations from intended intervention; D, missing outcome data; E, measurement of the outcome; F, selection of the reported result; and G, overall bias. Risk of bias arising from period and carryover effects was only applicable to crossover trials.

The pooled effect summary was calculated with the χ^2^ test. The test for group differences was calculated with meta-regression, which uses the Wald test.

†Post-hoc subgroup analysis by soy protein containing comparator and baseline BMI

*N=3 trial comparisons missing data for BMI

BMI, body mass index; CI, confidence interval; MD, mean difference

# **Supplementary Figure 15 (3 of 3).** Subgroup analyses for the effect of soy isoflavones on endometrial thickness

Pooled effect estimates for each subgroup and overall effect are represented by the diamonds. Data are expressed as weighted mean differences with 95% confidence intervals using the generic inverse-variance method and random effects DerSimonian-Laird model. Paired analyses were applied to all crossover trials. Inter‐study heterogeneity was assessed using the Cochrane Q statistic and quantified using the I^2^ statistic, with significance set at p<0.100 and I^2^≥50% considered to be evidence of substantial heterogeneity.

Risk of Bias Legend: (H) High Risk; (L) Low Risk; (S) Some concerns. The letters represent the following risk of bias domains: A, randomization process; B, bias arising from period and carryover effects; C, deviations from intended intervention; D, missing outcome data; E, measurement of the outcome; F, selection of the reported result; and G, overall bias. Risk of bias arising from period and carryover effects was only applicable to crossover trials.

The pooled effect summary was calculated with the χ^2^ test. The test for group differences was calculated with meta-regression, which uses the Wald test.

†Post-hoc subgroup analysis by type MD

CI, confidence interval; MD, mean difference

**Supplementary Figure 16 (1 of 3).** Subgroup analyses for the effect of soy isoflavones on follicle-stimulating hormone*

Pooled effect estimates for each subgroup and overall effect are represented by the diamonds. Data are expressed as weighted mean differences with 95% confidence intervals using the generic inverse-variance method and random effects DerSimonian-Laird model. Paired analyses were applied to all crossover trials. Inter‐study heterogeneity was assessed using the Cochrane Q statistic and quantified using the I^2^ statistic, with significance set at p<0.100 and I^2^≥50% considered to be evidence of substantial heterogeneity.

Risk of Bias Legend: (H) High Risk; (L) Low Risk; (S) Some concerns. The letters represent the following risk of bias domains: A, randomization process; B, bias arising from period and carryover effects; C, deviations from intended intervention; D, missing outcome data; E, measurement of the outcome; F, selection of the reported result; and G, overall bias. Risk of bias arising from period and carryover effects was only applicable to crossover trials.

The pooled effect summary was calculated with the χ^2^ test. The test for group differences was calculated with meta-regression, which uses the Wald test.

†Post-hoc subgroup analysis by continent

*N=6 trial comparisons missing data for years since menopause

^a^ Pairwise between-subgroup mean differences (95% CIs) for Continent were as follows: -14.8 (-21.6, -8.09) (4 vs 1); -10.9 (-19.1, -2.64) (4 vs 2); -11.8 (-20.7, -2.81) (4 vs 3); 19.7 (8.85, 30.6) (5 vs 4); 13.9 (4.86, 22.9) (6 vs 4)

^b^ Pairwise between-subgroup mean differences (95% CIs) for Intervention type were as follows: -18.1 (-30, -6.08) (2 vs 1); 19.9 (7.81, 32) (3 vs 2); 18.8 (0.377, 37.3) (4 vs 2)

CI, confidence interval; MD, mean difference

# **Supplementary Figure 16 (2 of 3).** Subgroup analyses for the effect of soy isoflavones on follicle-stimulating hormone*

Pooled effect estimates for each subgroup and overall effect are represented by the diamonds. Data are expressed as weighted mean differences with 95% confidence intervals using the generic inverse-variance method and random effects DerSimonian-Laird model. Paired analyses were applied to all crossover trials. Inter‐study heterogeneity was assessed using the Cochrane Q statistic and quantified using the I^2^ statistic, with significance set at p<0.100 and I^2^≥50% considered to be evidence of substantial heterogeneity.

Risk of Bias Legend: (H) High Risk; (L) Low Risk; (S) Some concerns. The letters represent the following risk of bias domains: A, randomization process; B, bias arising from period and carryover effects; C, deviations from intended intervention; D, missing outcome data; E, measurement of the outcome; F, selection of the reported result; and G, overall bias. Risk of bias arising from period and carryover effects was only applicable to crossover trials.

The pooled effect summary was calculated with the χ^2^ test. The test for group differences was calculated with meta-regression, which uses the Wald test.

†Post-hoc subgroup analysis by soy protein containing comparator and baseline BMI

*N=1 trial comparison missing data for BMI

^a^ Pairwise between-subgroup mean differences (95% CIs) for Energy control were as follows: -19.3 (-30.8, -7.67) (2 vs 1); 20.1 (7.88, 32.3) (3 vs 2)

BMI, body mass index; CI, confidence interval; MD, mean difference

# **Supplementary Figure 16 (3 of 3).** Subgroup analyses for the effect of soy isoflavones on follicle-stimulating hormone

Pooled effect estimates for each subgroup and overall effect are represented by the diamonds. Data are expressed as weighted mean differences with 95% confidence intervals using the generic inverse-variance method and random effects DerSimonian-Laird model. Paired analyses were applied to all crossover trials. Inter‐study heterogeneity was assessed using the Cochrane Q statistic and quantified using the I^2^ statistic, with significance set at p<0.100 and I^2^≥50% considered to be evidence of substantial heterogeneity.

Risk of Bias Legend: (H) High Risk; (L) Low Risk; (S) Some concerns. The letters represent the following risk of bias domains: A, randomization process; B, bias arising from period and carryover effects; C, deviations from intended intervention; D, missing outcome data; E, measurement of the outcome; F, selection of the reported result; and G, overall bias. Risk of bias arising from period and carryover effects was only applicable to crossover trials.

The pooled effect summary was calculated with the χ^2^ test. The test for group differences was calculated with meta-regression, which uses the Wald test.

†Post-hoc subgroup analysis by type MD

MD, mean difference; CI, confidence interval

# **Supplementary Figure 17 (1 of 3).** Subgroup analyses for the effect of soy isoflavones on estradiol*

Pooled effect estimates for each subgroup and overall effect are represented by the diamonds. Data are expressed as weighted mean differences with 95% confidence intervals using the generic inverse-variance method and random effects DerSimonian-Laird model. Paired analyses were applied to all crossover trials. Inter‐study heterogeneity was assessed using the Cochrane Q statistic and quantified using the I^2^ statistic, with significance set at p<0.100 and I^2^≥50% considered to be evidence of substantial heterogeneity.

Risk of Bias Legend: (H) High Risk; (L) Low Risk; (S) Some concerns. The letters represent the following risk of bias domains: A, randomization process; B, bias arising from period and carryover effects; C, deviations from intended intervention; D, missing outcome data; E, measurement of the outcome; F, selection of the reported result; and G, overall bias. Risk of bias arising from period and carryover effects was only applicable to crossover trials.

The pooled effect summary was calculated with the χ^2^ test. The test for group differences was calculated with meta-regression, which uses the Wald test.

†Post-hoc subgroup analysis by continent

*N=2 trial comparisons missing data for age, and N=6 trial comparisons for years since menopause

^a^ Pairwise between-subgroup mean differences (95% CIs) for Comparator were as follows: 47 (6.46, 87.6) (6 vs 1); 12.1 (0.585, 23.6) (5 vs 3); 35.2 (16.9, 53.5) (6 vs 3); 31.6 (13.9, 49.3) (6 vs 4); 23.1 (4.08, 42.1) (6 vs 5); -18.6 (-34.4, -2.68) (15 vs 5); -31.4 (-48.6, -14.3) (7 vs 6); -25.2 (-43.8, -6.66) (9 vs 6); -30 (-57, -3.01) (11 vs 6); -36 (-56.5, -15.4) (13 vs 6); -33.4 (-51.1, -15.8) (14 vs 6); -41.7 (-62.9, -20.4) (15 vs 6); -16.4 (-31.8, -1.09) (15 vs 9); -32.1 (-62.9, -1.36) (15 vs 12)

CI, confidence interval; MD, mean difference

# **Supplementary Figure 17 (2 of 3).** Subgroup analyses for the effect of soy isoflavones on estradiol*

Pooled effect estimates for each subgroup and overall effect are represented by the diamonds. Data are expressed as weighted mean differences with 95% confidence intervals using the generic inverse-variance method and random effects DerSimonian-Laird model. Paired analyses were applied to all crossover trials. Inter‐study heterogeneity was assessed using the Cochrane Q statistic and quantified using the I^2^ statistic, with significance set at p<0.100 and I^2^≥50% considered to be evidence of substantial heterogeneity.

Risk of Bias Legend: (H) High Risk; (L) Low Risk; (S) Some concerns. The letters represent the following risk of bias domains: A, randomization process; B, bias arising from period and carryover effects; C, deviations from intended intervention; D, missing outcome data; E, measurement of the outcome; F, selection of the reported result; and G, overall bias. Risk of bias arising from period and carryover effects was only applicable to crossover trials.

The pooled effect summary was calculated with the χ^2^ test. The test for group differences was calculated with meta-regression, which uses the Wald test.

†Post-hoc subgroup analysis by soy protein containing comparator and baseline BMI

*N=2 trial comparisons missing data for BMI

BMI, body mass index; CI, confidence interval; MD, mean difference

# **Supplementary Figure 17 (3 of 3).** Subgroup analyses for the effect of soy isoflavones on estradiol

Pooled effect estimates for each subgroup and overall effect are represented by the diamonds. Data are expressed as weighted mean differences with 95% confidence intervals using the generic inverse-variance method and random effects DerSimonian-Laird model. Paired analyses were applied to all crossover trials. Inter‐study heterogeneity was assessed using the Cochrane Q statistic and quantified using the I^2^ statistic, with significance set at p<0.100 and I^2^≥50% considered to be evidence of substantial heterogeneity.

Risk of Bias Legend: (H) High Risk; (L) Low Risk; (S) Some concerns. The letters represent the following risk of bias domains: A, randomization process; B, bias arising from period and carryover effects; C, deviations from intended intervention; D, missing outcome data; E, measurement of the outcome; F, selection of the reported result; and G, overall bias. Risk of bias arising from period and carryover effects was only applicable to crossover trials.

The pooled effect summary was calculated with the χ^2^ test. The test for group differences was calculated with meta-regression, which uses the Wald test.

†Post-hoc subgroup analysis by type MD

CI, confidence interval; MD, mean difference

# **Supplementary Figure 18.** Risk of bias subgroup analyses for the effect of soy isoflavones on endometrial thickness

Pooled effect estimates for each subgroup and overall effect are represented by the diamonds. Data are expressed as weighted mean differences with 95% confidence intervals using the generic inverse-variance method and random effects DerSimonian-Laird model. Paired analyses were applied to all crossover trials. Inter‐study heterogeneity was assessed using the Cochrane Q statistic and quantified using the I^2^ statistic, with significance set at p<0.100 and I^2^≥50% considered to be evidence of substantial heterogeneity.

MD, mean difference

# **Supplementary Figure 19.** Risk of bias subgroup analyses for the effect of soy isoflavones on follicle-stimulating hormone

Pooled effect estimates for each subgroup and overall effect are represented by the diamonds. Data are expressed as weighted mean differences with 95% confidence intervals using the generic inverse-variance method and random effects DerSimonian-Laird model. Paired analyses were applied to all crossover trials. Inter‐study heterogeneity was assessed using the Cochrane Q statistic and quantified using the I^2^ statistic, with significance set at p<0.100 and I^2^≥50% considered to be evidence of substantial heterogeneity. Note: Domain S refers to the risk of bias arising from period and carryover effects and was only applicable to crossover trials.

MD, mean difference

# **Supplementary Figure 20.** Risk of bias subgroup analyses for the effect of soy isoflavones on estradiol

Pooled effect estimates for each subgroup and overall effect are represented by the diamonds. Data are expressed as weighted mean differences with 95% confidence intervals using the generic inverse-variance method and random effects DerSimonian-Laird model. Paired analyses were applied to all crossover trials. Inter‐study heterogeneity was assessed using the Cochrane Q statistic and quantified using the I^2^ statistic, with significance set at p<0.100 and I^2^≥50% considered to be evidence of substantial heterogeneity. Note: Domain S refers to the risk of bias arising from period and carryover effects and was only applicable to crossover trials.

MD, mean difference

# **Supplementary Figure 21.** Continuous meta-regression analysis for the effect of soy isoflavones on endometrial thickness*

Data is presented as between group mean difference (95% CI) for a 1-unit change in the predictor variable. β –coefficients were estimated using continuous meta-regression analysis. A positive β -coefficient implies an increase in outcome in the isoflavone intervention as the subgroup variable increases, and a negative β -coefficient implies a decrease in outcome. Residual I^2^ reports inter-study heterogeneity not explained by the subgroup and was estimated using the Cochran Q statistic.

*N=2 trial comparisons did not report age, N=3 did not report years since menopause and baseline BMI

# **Supplementary Figure 22.** Continuous meta-regression analysis for the effect of soy isoflavones on follicle-stimulating hormone*

Data is presented as between group mean difference (95% CI) for a 1-unit change in the predictor variable. β –coefficients were estimated using continuous meta-regression analysis. A positive β -coefficient implies an increase in outcome in the isoflavone intervention as the subgroup variable increases, and a negative β -coefficient implies a decrease in outcome. Residual I^2^ reports inter-study heterogeneity not explained by the subgroup and was estimated using the Cochran Q statistic.

*N=6 trial comparisons did not report years since menopause, N=1 did not report baseline BMI

# **Supplementary Figure 23.** Continuous meta-regression analysis for the effect of soy isoflavones on estradiol*

Data is presented as between group mean difference (95% CI) for a 1-unit change in the predictor variable. β –coefficients were estimated using continuous meta-regression analysis. A positive β -coefficient implies an increase in outcome in the isoflavone intervention as the subgroup variable increases, and a negative β -coefficient implies a decrease in outcome. Residual I^2^ reports inter-study heterogeneity not explained by the subgroup and was estimated using the Cochran Q statistic.

* N=1 trial comparison did not report age, N=6 did not report years since menopause, and N=1 did not report baseline BMI

# **Supplementary Figure 24.** Linear and non-linear meta-regression analysis for the effect of soy isoflavones on endometrial thickness

Individual studies are represented by the circles, with their weight in the overall analysis represented by the size of the circles. The straight red line represents the estimate linear dose response and the grey line the non-linear dose response for the isoflavone dose and the dashed lines represent the upper and lower 95% confidence intervals.

ET, endometrial thickness

# **Supplementary Figure 25.** Linear and non-linear meta-regression analysis for the effect of soy isoflavones on vaginal maturation index

Individual studies are represented by the circles, with their weight in the overall analysis represented by the size of the circles. The straight red line represents the estimate linear dose response and the grey line the non-linear dose response for the isoflavone dose and the dashed lines represent the upper and lower 95% confidence intervals.

VMI, vaginal maturation index

# **Supplementary Figure 26.** Linear and non-linear meta-regression analysis for the effect of soy isoflavones on follicle-stimulating hormone

Individual studies are represented by the circles, with their weight in the overall analysis represented by the size of the circles. The straight red line represents the estimate linear dose response and the grey line the non-linear dose response for the isoflavone dose and the dashed lines represent the upper and lower 95% confidence intervals.

FSH, follicle-stimulating hormone

# **Supplementary Figure 27.** Linear and non-linear meta-regression analysis for the effect of soy isoflavones on estradiol

Individual studies are represented by the circles, with their weight in the overall analysis represented by the size of the circles. The straight red line represents the estimate linear dose response and the grey line the non-linear dose response for the isoflavone dose and the dashed lines represent the upper and lower 95% confidence intervals.

E2, estradiol

# **Supplementary Figure 28.** Publication bias funnel plots for the effect of soy isoflavones on endometrial thickness

Contour-enhanced funnel plot is a scatter-plot of each trial comparison weighted mean difference (MD) on the x-axis with the standard error (SE) representing precision on the y-axis. The vertical solid red line represents the pooled effect estimate and the dashed red lines represent the pseudo-95% confidence limits. The blue dots represent individual trial comparisons. The contour regions define the regions for the test of significance of individual study effect size for a given p-value range >0.1 (dark grey), 0.5 to <0.1 (medium grey), 0.01 to <0.5 (light grey), <0.01 (white)]. The contour-enhanced funnel plots may suggest funnel-plot asymmetry is due to publication bias when less precise (smaller) studies are missing in the non-significant regions. Quantitative assessment of publication bias was also performed using Egger's and Begg's tests set at a significance level of p<0.05.

CI, confidence interval

# **Supplementary Figure 29.** Publication bias funnel plots for the effect of soy isoflavones on follicle-stimulating hormone

Contour-enhanced funnel plot is a scatter-plot of each trial comparison weighted mean difference (MD) on the x-axis with the standard error (SE) representing precision on the y-axis. The vertical solid red line represents the pooled effect estimate and the dashed red lines represent the pseudo-95% confidence limits. The blue dots represent individual trial comparisons. The contour regions define the regions for the test of significance of individual study effect size for a given p-value range >0.1 (dark grey), 0.5 to <0.1 (medium grey), 0.01 to <0.5 (light grey), <0.01 (white)]. The contour-enhanced funnel plots may suggest funnel-plot asymmetry is due to publication bias when less precise (smaller) studies are missing in the non-significant regions. Quantitative assessment of publication bias was also performed using Egger's and Begg's tests set at a significance level of p<0.05.

CI, confidence interval

# **Supplementary Figure 30.** Publication bias funnel plots for the effect of soy isoflavones on estradiol

Contour-enhanced funnel plot is a scatter-plot of each trial comparison weighted mean difference (MD) on the x-axis with the standard error (SE) representing precision on the y-axis. The vertical solid red line represents the pooled effect estimate and the dashed red lines represent the pseudo-95% confidence limits. The blue dots represent individual trial comparisons. The contour regions define the regions for the test of significance of individual study effect size for a given p-value range >0.1 (dark grey), 0.5 to <0.1 (medium grey), 0.01 to <0.5 (light grey), <0.01 (white)]. The contour-enhanced funnel plots may suggest funnel-plot asymmetry is due to publication bias when less precise (smaller) studies are missing in the non-significant regions. Quantitative assessment of publication bias was also performed using Egger's and Begg's tests set at a significance level of p<0.05.

CI, confidence interval
